# Evaluations of effective coverage of maternal and child health services: A systematic review

**DOI:** 10.1093/heapol/czac034

**Published:** 2022-04-23

**Authors:** Aster Ferede Gebremedhin, Angela Dawson, Andrew Hayen

**Affiliations:** Department of Public Health, College of Health Sciences, Debre Markos University, PO Box 269, Debre Markos, Ethiopia; School of Public Health, University of Technology Sydney, PO Box 123, Broadway NSW 2007, Sydney, Australia; School of Public Health, University of Technology Sydney, PO Box 123, Broadway NSW 2007, Sydney, Australia; School of Public Health, University of Technology Sydney, PO Box 123, Broadway NSW 2007, Sydney, Australia

**Keywords:** Effective coverage, crude coverage, quality, maternal and child health, systematic review

## Abstract

Conventionally used coverage measures do not reflect the quality of care. Effective coverage (EC) assesses the extent to which health care services deliver potential health gains to the population by integrating concepts of utilization, need and quality. We aimed to conduct a systematic review of studies evaluating EC of maternal and child health services, quality measurement strategies and disparities across wealth quantiles. A systematic search was performed in six electronic databases [MEDLINE, EMBASE, Cumulative Index of Nursing and Allied Health (CINAHL), Scopus, Web of Science and Maternity and Infant Care] and grey literature. We also undertook a hand search of references. We developed search terms having no restrictions based on publication period, country or language. We included studies which reported EC estimates based on the World Health Organization framework of measuring EC. Twenty-seven studies, all from low- and middle-income settings (49 countries), met the criteria and were included in the narrative synthesis of the results. Maternal and child health intervention(s) and programme(s) were assessed either at an individual level or as an aggregated measure of health system performance or both. The EC ranged from 0% for post-partum care to 95% for breastfeeding. When crude coverage measures were adjusted to account for the quality of care, the EC values turned lower. The gap between crude coverage and EC was as high as 86%, and it signified a low quality of care. The assessment of the quality of care addressed structural, process and outcome domains individually or combined. The wealthiest 20% had higher EC of services than the poorest 20%, an inequitable distribution of coverage. More efforts are needed to improve the quality of maternal and child health services and to eliminate the disparities. Moreover, considering multiple dimensions of quality and the use of standard measurements are recommended to monitor coverage effectively.

Key messages

- Effective coverage (EC) metrics, integrating both the conventional measurement of crude coverage and quality of care, are critical for monitoring progress towards the universal health coverage with high-quality services.
- The EC of maternal and child health interventions lagged substantially behind crude coverage indicating a low quality of care.
- The wealthiest quintile had higher EC of services than the poorest quintile, showing an inequitable distribution of coverage.
- The results of the review also highlighted that the quality measurement strategies used often did not consider the multiple domains of quality of care, resulting in overestimated or underestimated coverage estimates.

## Introduction

Universal health coverage aims to provide access to promotive, preventive, curative and rehabilitative health services of adequate quality for people in need without financial hardship (Boerma *et al.*, 2018). The term ‘coverage’ defines the proportion of the population who require services that seek and receive these services (Bryce *et al.*, 2013). Coverage data provide an opportunity to monitor progress towards the sustainable development goals. Improved coverage and access to maternal and child health (MCH) services have the potential to reduce mortality and morbidity substantially (Leegwater *et al.*, 2015).

MCH remains a global health challenge. In 2017, the estimated maternal and infant mortality rates were 211 per 100 000 and 29 per 1000 live births, respectively (World Health Organization, 2019). There were approximately 5.5 million deaths among children under five years of age, in the same year (Hug *et al.*, 2019; 2018). Maximizing coverage is a key strategy to address these issues, but increasing coverage levels has not yet yielded the expected improvements and does not necessarily translate into better health gains (Marchant *et al.*, 2016). The persistence of preventable maternal and child deaths calls for better measures beyond the conventional coverage. Research suggests that poor quality of care may reduce the effectiveness of services provided (Souza *et al.*, 2013; Chou *et al.*, 2019). Increased access to and use of interventions provided to mothers and children may not be followed by encouraging outcomes if the quality of services is suboptimal. Despite the availability of greater access to maternity services, there is no strong evidence that demonstrates reduced maternal and neonatal mortality rates, which can be largely explained by the poor quality of care (Powell-Jackson *et al.*, 2015) (Okeke and Chari, 2015). Therefore, better MCH outcomes require improvements in the quality of care. The concept of quality is heavily debated. Donabedian’s quality framework is a widely adopted model in health care that considers quality as the combination of structural, process and outcome elements. A structural quality measure assesses characteristics of health care organizations or providers relevant to their capacity to provide optimal service to the population in need. Hence, it defines the environment in which health care is provided. Process elements of quality are defined by the activities or clinical actions that take place during the delivery of care to patients. The third component, an outcome measure, seeks to capture whether the goals of care were achieved. Each domain of quality measurement represents a piece of the complete picture but may not be used as the sole measure of quality (Donabedian, 1988).

Expanding the scope of the concept of quality care beyond access has led to an increased interest in the use of effective coverage (EC) as a comprehensive measure of the performance of a health system in a given setting, based on recommendations by the World Health Organization (WHO; World Health Organization, 2018). Measuring the performance of a health system is essential for identifying problems and improvements, supporting decision-making efforts and enabling successful policy formulation (Smith *et al.*, 2009). In view of this, EC is a suitable metric to provide a more nuanced understanding of whether, and how well, a health system is delivering services to its populations (Ng *et al.*, 2014). Quality-adjusted coverage, quality-adjusted contact or high-quality contact can be used alternatively to denote EC because quality is a major component of the metrics (Joseph *et al.*, 2020). The conventionally used measurement of crude coverage (CC), also called contact coverage, describes the utilization of services, but it does not consider the quality of care received. In contrast, EC integrates the concepts of need, use and quality into a single measure (Shengelia *et al.*, 2005). As a first framework, Tanahashi’s model of health service coverage introduced five stages of service provision, namely availability, accessibility, acceptability, contact or actual use and quality (Tanahashi, 1978). In search of a better integrating concept, the WHO’s updated framework further explained EC as the product of utilization and quality, conditional on the need for the service (Shengelia *et al.*, 2005). EC can be applied for one or a group of interventions or for the health system as a whole (Laurell, 2007).

A scoping review conducted by Jannati *et al.* assessed the key elements and steps of EC measurement, including the types of interventions covered and the strategies used to determine the constructs of EC (Jannati *et al.*, 2018). MCH interventions and chronic conditions were discussed. However, the scoping review has not offered a complete account of the specific estimates of the EC of MCH services, as well as the gaps and distributions across socio-economic status. Scoping reviews, which may be helpful precursors to systematic reviews, do not aim to produce a critically appraised and synthesized result for a particular question. In contrast, systematic reviews undergo an assessment of methodological limitations or risk of bias of evidence and provide evidence to inform practice (Munn *et al.*, 2018). Another comprehensive review of published literature on EC of reproductive, maternal, neonatal, child health and nutrition used evidence from Pubmed to discuss the gap between CC and quality-adjusted coverage (Amouzou *et al.*, 2019). In addition, this study developed a cascade framework with consecutive coverage steps to identify the potential losses of health benefits of interventions based on the Tanahashi framework (Tanahashi, 1978). Synthesizing evidence on the EC of MCH services systematically has a critical role in enhancing the foundation for assessing and improving progress. In this systematic review, we aimed to assess the EC of MCH interventions or programmes based on the updated WHO framework for measuring EC. We also investigated the gap between EC and CC, the quality measurement strategies used and possible disparities across different socio-economic groups. To our knowledge, this systematic review is the first to address these objectives.

## Methods

### Search strategy

This systematic review has been registered on the International Prospective Register of Systematic Reviews (PROSPERO CRD42020159384). Reporting has been done in accordance with the preferred reporting items for systematic reviews and meta-analyses guidelines (Moher *et al.*, 2009).

We conducted a comprehensive search of peer-reviewed literature in multiple electronic databases, including MEDLINE, CINAHL, EMBASE, Scopus, Web of Science and Maternity and Infant Care. In addition, grey literature from sources, including ProQuest, Google search engine, Google Scholar, Maternal Health Task Force and WHO’s official website, were searched for relevant reports and web-based publications. Moreover, the researcher manually searched the references of selected studies for relevant articles not identified in the initial search. Finally, we added new articles upon receiving search alerts from databases. Both free-text and Medical Subject Headings terms combined with Boolean operators, wildcards and truncations were included in the search strategy, including terms related to: mother, children, neonate, MCH services, antenatal care (ANC), post-natal care, family planning, delivery, management of childhood illnesses, immunization, neonatal care and EC. We developed the initial search strategy in OVID MEDLINE and adapted it for other databases (Supplementary_File_1).

### Eligibility criteria

Studies retrieved by the search strategy developed were exported to a citation manager (EndNote software) to accumulate relevant articles and to remove duplicates. The remaining articles were imported to Covidence where duplicates missed by the endnote were taken out. Next, we screened papers by reviewing the title and abstract and then by reviewing the full text.

All studies (prevalence studies, survey/national survey-based studies and other observational studies) that reported estimates of EC using the WHO framework of measuring EC were included (Shengelia *et al.*, 2005). During the search process, we identified no experimental or interventional studies. We considered both published and unpublished studies (grey literature) without restrictions based on publication period, country and language. Where studies were published in languages other than English, native language speakers were contacted for a translation. To be included in the review, the populations of interest should include at least one of the following: women aged 15–49 years, children under 5 years of age and neonates. We excluded qualitative studies, review articles, news items, commentaries, poster presentations, technical reports and editorials. In addition, we excluded studies that were not fully accessible after at least two-email contact with the primary authors due to the challenges to assess the quality of studies without full text. We excluded studies that used EC models other than the WHO model, such as the Tanahashi model, those that measured CC only (not adjusted for quality) or did not assess EC and studies that considered populations other than women aged 15 to 49 years and children under 5 years. The main outcome is EC, which is expressed as the product of CC (which consists of utilization and need components) and quality score.

### Quality assessment, data extraction and data analysis

We used the Joanna Briggs Institute critical appraisal checklist for prevalence studies to evaluate the methodological quality of the included studies. The tool consists of nine items that assess the internal and external validity of studies included in the qualitative analysis. During the quality appraisal, three authors were involved, ensuring each study was appraised by two authors, with any disagreements between authors resolved through discussion. Articles receiving a minimum score of 6 out of 9 were classified as having adequate quality. For each eligible article, information about author(s), the study setting, study period, aims, target population, intervention (programme) assessed and measurement strategy used to determine quality were extracted on Microsoft Excel 2016. In addition, a summary of results, including estimations of CC and EC, gaps between EC and CC and distributions of EC measures across different socio-demographic characteristics, were put into a predesigned summary table (see Table 1). We used a qualitative approach to summarize the characteristics of included studies and to synthesize the relevant information based on the objectives of the study. We discussed EC and CC estimates descriptively. We did not perform a meta-analysis of the findings due to the considerable heterogeneity of the included studies in terms of measurement methods used.

**Table 1.** EC and crude coverage estimates of maternal and child health services, the gaps and the distribution across wealth quintiles

No.	Author and country	Intervention (s)	EC (%)	Quality measurement domain	CC (%) and percentage gap between EC and CC	EC across wealth quintiles
1	(Hategeka *et al.*, 2020a), Rwanda	ANC Delivery care Care for child diarrhoea Care for child pneumonia Care for chid fever	Average EC has increased from 21% in 2010 to 33% in 2015 across all five services. EC was 20% for ANC, 40% for facility delivery, 44% for child pneumonia, 34% for child fever and 27% for child diarrhoea.	Processes of care (care competence, system competence and positive user experience)	Average CC has increased from 48% (27% gap) in 2010 to 57% (24% gap) in 2015. CC was 44% (24% gap) for ANC, 91% (51% gap) for facility delivery, 54% (10% gap) for child pneumonia, 50% (16% gap) for child fever and 44% (17% gap) for child diarrhoea.	EC remained largely inequitable across wealth quintiles.
2	(Nguhiu *et al.*, 2017a), Kenya	. Family planning (FP) ANC Skilled delivery & perinatal care Exclusive breast feeding (EBF) Immunization Management of Diarrhoea Care seeking for acute respiratory illness (ARI) Use of insecticide treated nets (ITN)	. Aggregate EC has increased from 27% in 2003 to 51% in 2014. In 2014, EC was 41% for FP, 45% for ANC, 51% for skilled delivery & perinatal care, 72% for EBF, 56% for immunization, 54% for management of Diarrhoea, 41% for care seeking for ARI, and 59% for use of ITN.	Processes of care	. Aggregate CC has increased from 45% (18% gap) in 2003 to 68% (17% gap) in 2014. In 2014, CC was 68% (27% gap) for FP, 58% (13% gap) for ANC, 61% (10% gap) for skilled delivery & perinatal care, 99.6% (28% gap) for EBF, 80% (24% gap) for immunization, 82% (28% gap) for management of diarrhoea, 59% (18% gap) for care seeking for ARI, and 75% (16% gap) for use of ITN.	. The wealthiest quintile had higher EC of services than the poorest quintile. With the increase in aggregate EC, there has been a general reduction in the economic inequalities in EC for MCH services.
3	(Joseph *et al.*, 2020a), Malawi	.Nutrition interventions during ANC & delivery	. Women attended a median of three ANC visits but received a median of 1.6 interventions on iron folic acid, 1 instance of counselling on diet during pregnancy, and 0.06 instances of counselling on optimal breastfeeding. Women thus received a median of 1.35 maternal nutrition interventions and 0.57 interventions that might increase uptake of breastfeeding.	Processes of care	Utilization of ANC and facility-delivery was high. After adjustment for nutrition-related quality, women received nutrition-related interventions considerably less often than they sought care.	–
4	(Lozano *et al.*, 2006a), Mexico	. ANC Skilled birth attendance (SBA) Services delivered to premature babies Treatment of ARI in children	ANC- 67 % SBA- 93 % Services delivered to premature babies- 81 % ARI- 58.1 %	Process of care & outcome	EC measures were lower than CC estimates.	. Inequalities exist in EC between income quintiles. The absolute gap in EC between quintiles is 9% for the MCH interventions.
5	(Yakob *et al.*, 2019a), Ethiopia	. ANC FP	ANC- 21.5% FP- 21.7%	Processes of care	CC was 62.4% (41% gap) for ANC, and 60.6% (39% gap) for FP.	–
6	(Gutiérrez, 2013a), Mexico	. ARI treatment Delivery care Prenatal care	EC ranged from 59% for ARI treatment to 94% for delivery care.	Processes of care	–	Care of ARI is significantly greater among non-poor in relation to the multidimensional poor and those who are vulnerable due to deficiencies, as well as the socio-economic quintile. The estimated gap between quintile I and V is 29.8%, which translates into coverage of more than 17 percentage points higher in quintile V. In the case of hospital care during childbirth, the differences are not significant, although a tendency to give greater coverage among those in better socio-economic conditions is also identified. The gap for adequate prenatal care is 27.1%, between a coverage of 72.4% in the first quintile, and 92.0% in the fifth.
7	(Martínez *et al.*, 2011a), Latin American & the Caribbean	.Breast feeding	EC ranged from 52% to 95%.	Development of acute diarrheal disease and ARI	CC results were given for the MCH interventions, but quality was not measured except for breast feeding.	–
8	(Wang *et al.*, 2019a), Bangladesh, Haiti, Malawi, Nepal, Senegal, Tanzania	Facility delivery	EC was 26.8 % in Bangladesh, 24.4 % in Haiti, 66.4% in Malawi, 41.9% in Nepal, 51.3% in Senegal and 44.2% in Tanzania.	Structure	CC was 39.7% (13% gap) in Bangladesh, 40% (16% gap) in Haiti, 92.9% (27% gap) in Malawi, 52.7% (11% gap) in Nepal, 77% (26% gap) in Senegal, and 65% (21% gap) in Tanzania.	–
9	(Nesbitt *et al.*, 2013a), Ghana	SBA	EC was 18%.	Processes of care & structure	.CC was 68% (50% gap)	- EC varied with wealth quintile; 4% of live-births in the lowest wealth quintile were in high quality facilities compared to 37% of live-births in the highest quintile.
10	(Koulidiati *et al.*, 2018a), Burkina Faso	Care seeking for childhood illnesses	EC was 5.3% considering high quality, and 44.6% (Considering both high and intermediate quality facilities).	Processes of care & structure	CC was 69.5% (64% gap considering high-quality facilities).	–
11	(Leslie *et al.*, 2017a), Haiti, Kenya, Malawi, Namibia, Senegal, Rwanda, Tanzania, Uganda	ANC FP Care for seek children < 5	. Average EC of the three services was 25.4% across all countries. Individually, the average EC across these services was: 19.2% in Haiti, 26.3% in Kenya, 24.7% in Malawi, 40.7% in Namibia, 19% in Senegal, 24.5% in Rwanda, 22.5% in Tanzania, and 26.3% in Uganda.	Processes of care	. Average CC of the three services was 69.2% (44% gap) across all countries. Individually, the average CC across these services was: 58.9% in Haiti, 67.1% in Kenya, 81.2% in Malawi, 80.3% in Namibia, 58.8% in Senegal, 67.5% in Rwanda, 67.5% in Tanzania, and 68.2% in Uganda.	EC was highest on average in Namibia (by far the wealthiest country in the sample).
12	(Carter *et al.*, 2018a), Zambia	Care for sick children <5	Using exact-match linking: EC of was estimated at 60% in the rural area and 49% in the urban area.	Structure	From the exact-match linking result: There was a16-point rural gap and 13-point urban gap in coverage between seeking skilled care and EC. (i.e.CC was 76% in the rural area and 62% in the urban area).	–
13	(Leslie *et al.*, 2019a), Mexico	ANC Delivery care Newborn care Under 5 diarrhoea	EC was 63.3% for ANC, 31.1% for delivery care, 74.3% for newborn care and 26.8% for under-5 diarrhoea	Outcome	–	Substantial inequality in EC existed between states, but wealth status was not considered.
14	(Marchant *et al.*, 2015a), Nigeria, Ethiopia, and India	ANC SBA Post-partum checks (PPC) Post-natal care (PNC)	In Gombe, EC was 11% for ANC, 8% for SBA, 0% for PPC, and 0% for PNC. In Ethiopia, EC was 4% for ANC, 4% for SBA, 0% for PPC, and 0% for PNC. In India, EC was 6% for ANC, 4% for SBA, 0% for PPC, and 0% for PNC.	Processes of care	In Gombe, CC was 61% for ANC, 22% for SBA, 7% for PPC, and 4% for PNC. In Ethiopia, CC was 56% for ANC, 15% for SBA, 3% for PPC, and 4% for PNC. In India CC was 74% for ANC, 76% for SBA, 54% for PPC, and 19% for PNC. The gap ranges from 3% for post-partum check (Ethiopia) to 72% for SBA (India).	–
15	(Okawa *et al.*, 2019a), Myanmar	ANC Peripartum care and PNC	The EC was 14.6% for ANC, 15.2% for peripartum care and 3.6% for PNC.	Processes of care	The CC was 60.9% (46% gap) for ANC, 61.3% (46% gap) for peripartum care and 11.5% (8% gap) for PNC.	Household wealth was not associated with receiving high-quality care.
16	(Murphy *et al.*, 2018a), Kenya	Inpatient neonatal care	EC was 25%.	Structure & processes of care	–	–
17	(Willey *et al.*, 2018a), Uganda	SBA	. Using the individual-linking method, EC of SBA was 10%. Using ecological linking method EC ranged from 4.68 % to 11 %.	Structure	CC was 55% (45% gap when using the individual linking method & 44% to 50% when using ecological linking method).	–
18	(Larson *et al.*, 2017a), Tanzania	Delivery care	.EC was 25%. However, applying a conservative standard (90% completion of required elements), the EC was zero.	Structure & processes of care	CC was 82% (57% gap).	The wealthiest 20% of women were 4.1 times as likely to deliver in facilities offering at least the minimum threshold of quality care through the cascade compared to the poorest 80% of women. EC of delivery care was very low, particularly among poorer women.
19	(Munos *et al.*, 2018a), Côte d’Ivoire	ANC Delivery and immediate newborn care PNC Care seeking for sick child	EC estimates generated using ecological and exact-match linking methods varied across the interventions.	Structure & processes of care	.CC was 82% for ANC, 65% for delivery care, 65% for newborn care, 5% for PNC, and 43% for sick childcare. EC estimates computed using exact-match methods were 13%-63% lower than the care seeking estimates from the CC.	–
20	(Hodgins and D’agostino, 2014), 41 countries	ANC	EC ranged from 14% in Niger to 84% In Dominican republic.	Processes of care	Coverage for specific interventions was generally much lower among all pregnant women (reflecting population EC) than among only those who had received ANC 4+ visits.	–
21	(Idzerda *et al.*, 2011a), Roma, Serbia	ARI in children	EC was 36.8% in the general population, 30.8% in Roma and 66.7% in the poorest 20% of the Roma population.	Processes of care	CC was 63.2% (26% gap) in the general population, 67.4% (37% gap) in Roma, and 88.9% (22% gap) in the poorest 20%.	–
22	(Engle-Stone *et al.*, 2015a), Cameroon	Nutrition interventions	EC estimates varied across the nutrition interventions and they were lower.	Biomarkers	Estimates of coverage were greater than the EC estimates.	–
23	(Colston, 2011), Mexico & Nicaragua	Immunization	EC was 68% in Mexico and 50% in Nicaragua.	Biomarkers	CC was 83% (15% gap) in Mexico and 85% (35% gap) in Nicaragua.	–
24	(Venkateswaran *et al.*, 2019a), West Bank, Palestine	ANC	EC of the specific ANC interventions varied from 7% to 59%.	Processes of care	Coverage of one screening (conceptually equivalent to CC) and EC of ANC interventions were notably different for screening for: hypertension (98% vs. 10%); foetal growth abnormalities (66% vs. 6%); anaemia (93% vs. 14%); gestational diabetes (93% vs. 34%), and antenatal ultrasound (74% vs. 24%).	–
25	(Kyei *et al.*, 2012a), Zambia	ANC	Only 29% of mothers received good quality ANC and only 8% received good quality ANC and attended in the first trimester (as a proxy for EC).	Processes of care	94% (86% gap) of mothers had at least one ANC visit with a skilled health worker and 60% (52% gap) had at least four visits.	–
26	(Travassos *et al.*, 2016a), Ethiopia	Immunization	Estimates of immunization coverage by immunization card, maternal recall and protective serologic biomarkers varied across the study regions.	Biomarkers	- Among toddlers, the estimation of coverage based on documented vaccination (vaccination card or EPI registry record) was only slightly lower (4–11%) than the prevalence of protective tetanus antitoxin biomarkers. Moreover, among the toddlers whose evidence of vaccination derived from maternal recall, the prevalence of protective serologic biomarkers was higher than maternal recall estimates of coverage. - Estimates of pentavalent coverage by immunization card were lower than the prevalence of protective serologic biomarkers. In contrast, EPI register record estimates were similar to biomarker findings.	–
27	(Nguyen *et al.*, 2021a), Bangladesh	Nutrition interventions across the continuum of maternal and early childhood care (specifically ANC and delivery for women and growth monitoring and curative care for young children.	18% for ANC, 23% for institutional delivery, 20% for child growth monitoring and 52% for sick child Care.	Structure	Contact coverage varied from 28% for attending at least four ANC visits to 38% for institutional delivery, 35% for child growth monitoring and 81% for sick child care. - The gaps between contact and Input-adjusted coverage ranged between 10 and 30 percentage points.	Inequalities in input-adjusted coverage were large during ANC and institutional delivery (14–17 percentage points (pp) between urban and rural areas, 15 pp between low and high education, and 28-34 pp between highest and lowest wealth quintiles), but narrower for child growth monitoring and sick child care (<2 pp).

ARI, acute respiratory infections; EBF, exclusive breastfeeding; FP, family planning; ITN, insecticide-treated nets; PNC, post-natal care, PPC, post-partum care; SBA, skilled birth attendance.

## Results

**Figure 1.**
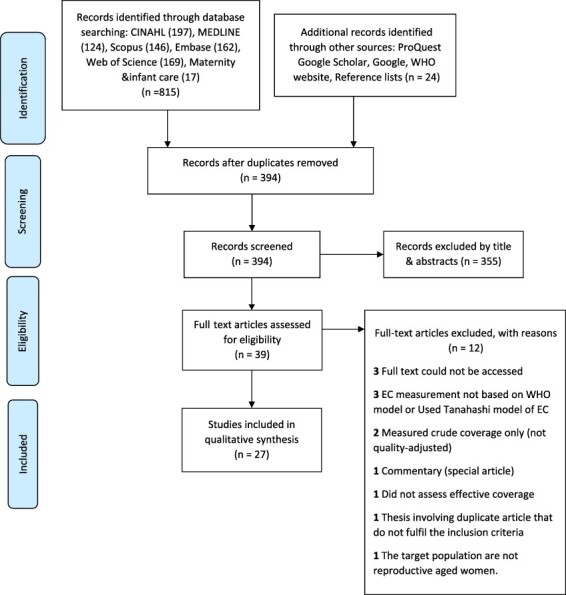
Preferred reporting items for systematic reviews and meta-analyses study selection flow diagram

A total of 839 citations were identified, leaving 394 unique studies after duplicates were removed. Three hundred fifty-five articles were excluded based on title and abstract. The remaining 39 articles were reviewed using the full text. Subsequently, we included 27 studies in the review after applying the inclusion and exclusion criteria (Figure 1).

### Characteristics of included studies

The 27 included studies were published between 2006 and 2021. Two of the studies (Gutiérrez, 2013; Martínez *et al.*, 2011) were reported in Spanish, while the rest were reported in English. Although we did not restrict studies by country, all of the included studies were conducted in low- and middle-income countries, where maternal and child mortality remains a major challenge to health systems. While the majority of studies were conducted in Sub-Saharan Africa (*n *= 14), 10 Latin-American countries (*n *= 5)3 and three Asian countries (*n *= 4) were represented. Four studies used data from multiple country settings (Wang *et al.*, 2019; Leslie *et al.*, 2017; Marchant *et al.*, 2015; Hodgins and D’Agostino, 2014). The predominant data sources used by the studies were Demographic and Health Surveys solely or combined with Service Provision Assessments (*n *= 9) (Nguhiu *et al.*, 2017; Yakob *et al.*, 2019; Wang *et al.*, 2019; Martínez *et al.*, 2011; Leslie *et al.*, 2017; Hodgins and D’Agostino, 2014; Kyei *et al.*, 2012; Hategeka *et al.*, 2020; Nguyen *et al.*, 2021). Demographic and Health Surveys are nationally representative household surveys that provide data for a wide range of monitoring and impact evaluation indicators in the areas of population, health and nutrition. The Service Provision Assessment survey is a health facility assessment that provides a comprehensive overview of a country’s health service delivery by collecting information on the overall availability of different facility-based health services and their readiness to provide those services. Two studies (Joseph *et al.*, 2020; Munos *et al.*, 2018) used Multiple Cluster Indicator Surveys, which were implemented by the United Nations Children’s Fund to provide internationally comparable data on the situation of children and women. The other data sources used in the reviewed articles include National Health and Nutrition Surveys (*n *= 3; Lozano *et al.*, 2006; Gutiérrez, 2013; Leslie *et al.*, 2019), surveillance data (*n *= 1; Nesbitt *et al.*, 2013), baseline surveys of government-led evaluations (*n *= 1; Koulidiati *et al.*, 2018), facility document reviews (*n *= 2; Murphy *et al.*, 2018; Venkateswaran *et al.*, 2019), household surveys (including health worker and facility assessments; *n *= 7; Carter *et al.*, 2018; Marchant *et al.*, 2015; Willey *et al.*, 2018; Larson *et al.*, 2017; Engle-Stone *et al.*, 2015; Colson *et al.*, 2015; Okawa *et al.*, 2019), immunization coverage surveys (*n *= 1; Travassos *et al.*, 2016) and reports from international organizations such as the World Bank and UNICEF (*n *= 1; Idzerda *et al.*, 2011).

### Quality appraisal result

All studies (100%) described the study subjects and the setting in detail. The majority of studies (96%) used an adequate sample size and 93% measured the outcome in a standard, reliable way for all participants. Eighty-nine per cent of the studies used an appropriate sampling frame and 24 studies (89%) sampled participants in an appropriate way. It was also noted that all studies had an adequate response rate and 85% used appropriate statistical techniques. However, valid methods for the identification of the outcome were not followed in seven studies (26%). Five studies (19%) did not conduct data analysis with sufficient coverage of the identified samples (Supplementary_File_2).

When we removed the two articles of lower quality (Martínez *et al.*, 2011; Idzerda *et al.*, 2011), we found that the extreme value (maximum EC) reported would be 94% for delivery care (Mexico; Gutiérrez, 2013) instead of 95% for breastfeeding (Dominican Republic) (Martínez *et al.*, 2011). However, the most important findings of the review, including the size of the gap between EC and CC, and the conclusions remained the same. We included all studies irrespective of the critical appraisal result.

### MCH interventions assessed

Research on EC has been undertaken with the aim of evaluating health system performance to deliver MCH services or to assess specific intervention(s) or programme(s). Four of the included articles calculated EC for key interventions at an individual level and combined these into composite metrics for health system assessment (Nguhiu *et al.*, 2017; Leslie *et al.*, 2019; 2017; Lozano *et al.*, 2006). The choice of indicators was based on a country’s needs and set priorities. Lozano *et al.* used EC as a performance-benchmarking device across states in Mexico (Lozano *et al.*, 2006a). They created an overall EC score combining 14 interventions, out of which 8 were for MCH, at the national level. Similarly, Nguhiu *et al.* constructed an aggregated EC measure out of eight MCH interventions weighted by population need for the services (Nguhiu *et al.*, 2017). Furthermore, Leslie *et al.* (Leslie *et al.*, 2017) quantified EC for three essential primary care services, namely ANC, family planning and care for sick children, across multiple countries and calculated primary care coverage by averaging these three services. A recent study by Leslie *et al.* described the EC of multiple conditions within the Mexican Institute of Social Security, the largest health system in Mexico, using routinely collected data and focusing on metrics of potential health gain or loss (Leslie *et al.*, 2019).

Papers that focused on the assessment of specific intervention(s) or programme(s) evaluated EC of maternal health services at pre-pregnancy (Nguhiu *et al.*, 2017; Yakob *et al.*, 2019; Leslie *et al.*, 2017), during pregnancy (Nguhiu *et al.*, 2017; Joseph *et al.*, 2020; Lozano *et al.*, 2006; Yakob *et al.*, 2019; Leslie *et al.*, 2017; 2019; Marchant *et al.*, 2015; Gutiérrez, 2013; Okawa *et al.*, 2019; Munos *et al.*, 2018; Hodgins and D’Agostino, 2014; Venkateswaran *et al.*, 2019; Kyei *et al.*, 2012), delivery (Nguhiu *et al.*, 2017; Lozano *et al.*, 2006; Wang *et al.*, 2019; Gutiérrez, 2013; Martínez *et al.*, 2011; Nesbitt *et al.*, 2013; Leslie *et al.*, 2019; Marchant *et al.*, 2015; Okawa *et al.*, 2019; Willey *et al.*, 2018; Larson *et al.*, 2017; Munos *et al.*, 2018; Hategeka *et al.*, 2020) and post-natal phases (Marchant *et al.*, 2015; Okawa *et al.*, 2019). Some articles reported the EC of individual elements of care during the delivery of interventions such as ANC (Hodgins and D’Agostino, 2014; Venkateswaran *et al.*, 2019a). The EC of child health interventions such as immunization (Travassos *et al.*, 2016a; Colson *et al.*, 2015; Nguhiu *et al.*, 2017), care-seeking for childhood illnesses (Hategeka *et al.*, 2020; Nguhiu *et al.*, 2017; Lozano *et al.*, 2006; Gutiérrez, 2013; Koulidiati *et al.*, 2018; Leslie *et al.*, 2017; Carter *et al.*, 2018; Leslie *et al.*, 2019; Munos *et al.*, 2018; Idzerda *et al.*, 2011) and breastfeeding (Nguhiu *et al.*, 2017; Martínez *et al.*, 2011) was also the other area of focus. Moreover, adjusted coverage measures for neonatal and infant health interventions were identified (Nguhiu *et al.*, 2017; Lozano *et al.*, 2006; Martínez *et al.*, 2011; Leslie *et al.*, 2019; Marchant *et al.*, 2015; Okawa *et al.*, 2019; Murphy *et al.*, 2018; Munos *et al.*, 2018). Three studies estimated the EC of nutrition interventions delivered to women and children (Joseph *et al.*, 2020; Engle-Stone *et al.*, 2015; Nguyen *et al.*, 2021). Overall, the large majority of included studies assess concerning to ANC and delivery care.

Drawing on EC framework, the reviewed studies calculated EC of MCH services as: EC = ‘Quality’ of MCH services × (‘Utilization’ of MCH services divided by ‘Need’ for MCH services). The following are some examples of how EC was defined. Okawa *et al.* defined the EC of ANC as the proportion of women receiving four or more visits (indicating coverage) and receiving 11–14 of the intervention items (indicating quality; Okawa *et al.*, 2019). Nesbitt *et al.* (2013) defined EC of delivery care as the proportion of deliveries in facilities offering high-quality care in all four quality dimensions (routine delivery, emergency obstetric care, emergency newborn care and non-medical quality). Koulidiati *et al.*, (2018) defined EC of care-seeking for child illness as the proportion of all under-5-year-old children in need who actually sought care at a facility categorized as of high quality. Colson *et al.* (2015) defined EC of measles immunization as the proportion of children with a positive dried blood sample assay for measles-specific Immunoglobulin G antibodies. Leslie *et al.* (2017) defined the EC of family planning as the proportion of women using modern contraceptive methods and receiving the essential clinical actions (16 items) within reproductive history, counselling and history and physical examination domains. Murphy *et al.* (2018) defined the EC of neonatal care as the proportion of newborns attending facilities providing high-quality care.

### EC estimates and the gaps with CC

The EC estimates extracted from the included studies ranged from 0% for post-partum care in Gombe in Nigeria, Ethiopia and Uttar Pradesh in India (Marchant *et al.*, 2015) to 95% for breastfeeding in the Dominican Republic (Martínez *et al.*, 2011). Studies that evaluated family planning showed that EC estimates lagged substantially behind the CC for the same service due to low-quality care. Supporting this evidence was the drop in EC from 68% to 41% in Kenya (Nguhiu *et al.*, 2017) and 61% to 22% in Ethiopia (Yakob *et al.*, 2019) when CC measures were adjusted with quality of care. Leslie *et al.* reported the average EC of family planning across eight countries that was estimated at 26%, ranging from 17% in Haiti to 38% in Rwanda (Leslie *et al.*, 2017). In all these countries, CC considerably overstated EC.

Twelve studies (Nguhiu *et al.*, 2017; Joseph *et al.*, 2020; Lozano *et al.*, 2006; Yakob *et al.*, 2019; Leslie *et al.*, 2017; 2019; Marchant *et al.*, 2015; Gutiérrez, 2013; Okawa *et al.*, 2019; Munos *et al.*, 2018; Kyei *et al.*, 2012; Hategeka *et al.*, 2020) showed that estimates of ANC would also be lower if the quality of care at facilities was taken into account. The gap between EC and CC was as high as 86% in Zambia (Kyei *et al.*, 2012). In this study, 94% of women had at least one ANC visit, while 60% had at least four visits. However, only 8% of mothers attended high-quality ANC and had their visit in the first trimester, hence denoting low EC.

In contrast to studies that assessed the provision of interventions at one time only, Venkateswaran *et al.* examined the appropriate number and timing of screening tests, simultaneously, for eight specific ANC interventions (such as screening for hypertension, anaemia and gestational diabetes) throughout the pregnancy period as a proxy for EC (Venkateswaran *et al.*, 2019). Accordingly, the EC of seven of these specific interventions was lower than the coverage of at least one screening and coverage of the appropriate number of screenings, both denoting CC measures. Hodgins *et al.* assessed population EC by taking the average coverage of specific interventions (a set of key antenatal services) among all pregnant women (Hodgins and D’Agostino, 2014). This contrasts with the measure of conventional coverage defined as the average coverage of specific interventions among those who had received four or more ANC visits. The results show coverage for specific interventions was generally much lower among all pregnant women (as a proxy for EC) than among only those who had received ANC four or more visits. The population EC ranged from 14% in Niger to 84% in the Dominican Republic.

For delivery and perinatal care, the highest gap between contact coverage and high-quality contact was 72% (in India, where the CC was 76% and the EC was 4%; Marchant *et al.*, 2015). Lozano *et al.* determined the EC of skilled birth attendance to be 93%, taking births that took place in hospitals as a quality-measuring indicator (Lozano *et al.*, 2006). In the articles that reported both coverage measures, it was found that adjusting for quality of care substantially reduced CC of delivery care (Nguhiu *et al.*, 2017; Wang *et al.*, 2019; Nesbitt *et al.*, 2013; Marchant *et al.*, 2015; Okawa *et al.*, 2019; Willey *et al.*, 2018; Larson *et al.*, 2017; Hategeka *et al.*, 2020).

When analysing the findings of a study on post-partum care coverage, it was found that quality-adjusted contact was considerably lower than the crude contact estimates. Seven per cent, 3% and 54% of women received post-partum care in Gombe, Ethiopia and Uttar Pradesh, respectively (Marchant *et al.*, 2015). However, high-quality post-partum care was zero in all these settings. In Myanmar, the coverage of peripartum care was 61%, but it dropped to 15% when quality was included in the metrics, denoting a 46% gap (Okawa *et al.*, 2019).

Few studies discussed nutrition-related interventions as a health system performance indicator. Joseph *et al.* adjusted coverage measures of ANC and delivery for quality of nutrition interventions (Joseph *et al.*, 2020). Results showed that after considering quality, women received nutrition interventions less often than they sought care. Related to this, a study in Cameroon (Engle-Stone *et al.*, 2015) measured the EC of nutrition programmes by taking the proportion of the population that had an inadequate nutrition intake at baseline and achieved a sufficient level following a given intervention. The EC estimates were lower than raw coverage (a proportion that is deficient and had received a programme) estimates almost by half. Nguyen *et al.* specifically examined nutrition interventions across the continuum of maternal and child care and found that coverage was 28% for ANC, 38% for institutional delivery, 35% for child growth monitoring and 81% for sick child care (Nguyen *et al.*, 2021). However, quality reduced the estimates to 18% for ANC, 23% for institutional delivery, 20% for child growth monitoring and 52% for sick childcare. This indicates there was a 10–30 percentage gap between the two estimates.

Combined facility and household surveys evaluated the coverage of various child health interventions in diverse settings and found that EC estimates were lower than CC estimates. For immunization, the CC vs EC results were 80% vs 56%, 83% vs 68% and 85% vs 50% in Kenya (Nguhiu *et al.*, 2017), Mexico and Nicaragua, (Colson *et al.*, 2015), respectively. In contrast, Travassos *et al.* linked serosurveys (where specimens obtained from selected populations are tested for antibodies) to immunization coverage surveys to measure the proportion of children protected against vaccine-preventable diseases in three regions in Ethiopia (Travassos *et al.*, 2016). Coverage measures from vaccination cards, immunization clinic records and maternal recall were compared with serosurveys. They found inaccuracies in administrative estimates of vaccine coverage, which over-estimated coverage where immunization services were weak. The authors highlighted, by detecting protective serologic biomarkers, that serosurveys are able to monitor objectively the proportion of children that have received a vaccine. This approach can best determine the quality of the immunization services and provide critical insights into the effectiveness of vaccination programmes.

CC for acute respiratory illnesses and diarrhoea and the corresponding EC estimates were notably different across countries. EC ranged from 5.3% in Burkina Faso (Koulidiati *et al.*, 2018; considering only those who received high-quality services) to 67% in Serbia (Idzerda *et al.*, 2011), and among the studies that reported both estimates (Hategeka *et al.*, 2020; Nguhiu *et al.*, 2017; Koulidiati *et al.*, 2018; Leslie *et al.*, 2017; Carter *et al.*, 2018; Idzerda *et al.*, 2011), coverage values became lower when adjusted for quality. The difference between the two coverage estimates was as high as 64 percentage points (Koulidiati *et al.*, 2018). Two of the included studies discussed breastfeeding. Nguhiu *et al.* (2017) found that coverage dropped from 99.6% to 72% when quality was taken into account, while Martínez *et al.* (2011) described that EC ranged from 52% to 95%. Seven of the studies identified by this review have discussed infant and neonatal interventions (Lozano *et al.*, 2006; Martínez *et al.*, 2011; Leslie *et al.*, 2019; Marchant *et al.*, 2015; Okawa *et al.*, 2019; Murphy *et al.*, 2018; Munos *et al.*, 2018). When quality was taken into account, post-natal care coverage decreased from 4%, 4% and 19% in Gombe state (Nigeria), Ethiopia and Uttar Pradesh (India), respectively, to 0% (Marchant *et al.*, 2015), whereas, in Myanmar, there was an eight-percentage difference between CC and EC (Okawa *et al.*, 2019). It was noted that EC of newborn care was 81% in Mexico (Lozano *et al.*, 2006), 25% in Kenya (Murphy *et al.*, 2018) and 74% in another study from Mexico (Leslie *et al.*, 2019), although they did not specify CC values (Supplementary_File_3).

### Quality measurement strategies

Researchers used various methods and strategies to measure the quality component of EC across different types of interventions. The bulk of studies used measures assessing the process of care (Hategeka *et al.*, 2020; Nguhiu *et al.*, 2017; Joseph *et al.*, 2020; Yakob *et al.*, 2019; Gutiérrez, 2013; Leslie *et al.*, 2017; Marchant *et al.*, 2015; Okawa *et al.*, 2019; Hodgins and D’Agostino, 2014; Idzerda *et al.*, 2011; Venkateswaran *et al.*, 2019; Kyei *et al.*, 2012). In this case, a list of recommended clinical actions that are performed during contact between health care users and a provider was used to measure quality. We identified themes under the process of care domain that were used to measure the quality of MCH interventions. These included a list of clinical and women-/children-centred activities conducted during history taking, physical examination, screening, preventive measures and counselling. For immunization, questions related to the use of guidelines and documentation of service delivery represented the process of care domain. Assessing quality using a checklist of services provided during birth such as active management of the third stage of labour also characterized the process of care.

Four studies used the structural aspect of quality of care, which incorporated observations of the physical attributes of a health facility including infrastructure, equipment, supplies, commodities and the availability of trained personnel (Wang *et al.*, 2019; Carter *et al.*, 2018; Willey *et al.*, 2018; Nguyen *et al.*, 2021). In other words, they assessed service or facility readiness to provide a particular intervention. Five of the included articles used combinations of structural and process domains (Nesbitt *et al.*, 2013; Koulidiati *et al.*, 2018; Murphy *et al.*, 2018; Larson *et al.*, 2017; Munos *et al.*, 2018). In contrast, Lozano *et al.* combined process and outcome domains of quality metrics (Lozano *et al.*, 2006). Other studies measured quality using an outcome domain such as risk-adjusted mortality (Leslie *et al.*, 2019; Lozano *et al.*, 2006). Three among the 27 included articles have shown that the presence of biomarkers, which are indicators of a particular disease condition or some other physiological state, can provide objective insights on the quality of immunization and nutrition services (Colson *et al.*, 2015; Travassos *et al.*, 2016; Engle-Stone *et al.*, 2015). Due to limitations presented on the estimation of the quality of interventions, some studies reported only CC values (Lozano *et al.*, 2006; Martínez *et al.*, 2011; Gutiérrez, 2013). However, these three studies were included because they have reported the results of EC at least for one MCH intervention. In this regard, Lozano *et al.* (2006) evaluated eight MCH interventions, but only four of them had EC values (Lozano *et al.*, 2006). Similarly, Gutiérrez (2013) evaluated five MCH interventions, although only three had EC values reported (Gutiérrez, 2013). Out of six interventions, Martínez *et al.* (2011) reported the EC of only breastfeeding (Martínez *et al.*, 2011; Supplementary_File_4).

### EC estimates across the wealth quintiles

Among the included studies, seven revealed that there were variations in EC estimates across different wealth quintiles (Hategeka *et al.*, 2020; Nguhiu *et al.*, 2017; Lozano *et al.*, 2006; Gutiérrez, 2013; Larson *et al.*, 2017; Nguyen *et al.*, 2021; Nesbitt *et al.*, 2013). Their finding indicated that the wealthiest quintile had a higher EC of services than the poorest quintile. Nguhiu *et al.* found that overall increases in EC of MCH services have occurred concomitantly with a drop in the levels of inequalities in EC over time (Nguhiu *et al.*, 2017). Inequalities were highest for delivery care, where the wealthiest quintile had three times greater EC than the poorest quintile. Significant inequalities in the EC of immunization, ANC and family planning have also been reported to the disadvantage of low-income people, showing that inequalities were particularly evident in maternal health services. The EC of management of diarrhoea exhibited a pro-poor distribution. In Rwanda, the increases in EC of ANC and delivery care were associated with widening inequality. EC improvements were greatest amongst wealthier quintiles of the population. Conversely, sick childcare showed narrowing inequalities in EC (Hategeka *et al.*, 2020). Lozano *et al.* (2006) estimated the absolute gap in EC between quintiles to be 9% for MCH services. On the other hand, Larson *et al.* found that compared to the poorer 80% of women, the wealthiest 20% of women were more likely to deliver in a good-quality facility and receive good-quality care (Larson *et al.*, 2017). Similarly, EC varied across wealth quintiles, where 4% of skilled deliveries in the lowest wealth quintile were in high-quality facilities compared to 37% of deliveries in the highest quintile (Nesbitt *et al.*, 2013). According to a recent study in Bangladesh, inequalities in nutrition input-adjusted coverage were large during ANC and institutional delivery as evidenced by the 28–34 percentage point difference between the highest and lowest wealth quintiles (Nguyen *et al.*, 2021). A study in Mexico reported that care-seeking for acute childhood illnesses was greater among the wealthy compared to low-income people (Gutiérrez, 2013). In contrast to the above studies, Okawa *et al.* determined that household wealth was not associated with receiving high-quality care (Okawa *et al.*, 2019).

## Discussion

Evidence indicates that expanding health care coverage does not necessarily result in better outcomes (Marchant *et al.*, 2016; Winter *et al.*, 2017). The persistent problems of high maternal, newborn and child morbidity and mortality require a functional health care system, which does not depend on the coverage of health services alone. CC estimates do not capture quality and hence do not show potential health gain. EC denotes the relationship between service utilization conditional on true need and the service quality received. It adjusts crude population-level coverage for quality of care thereby revealing gaps in the delivery of effective care (Colston, 2011). This systematic review revealed four major findings regarding EC of MCH services. First, most studies found lower EC estimates. Second, in all studies that reported both estimates, EC values were lower than the CC and there was a major difference between the two. Third, the quality component of EC was often measured using structure, process and outcome domains, but combinations of these have been used as well. Fourth, studies that compared EC across different socio-economic statuses found that the poorest quantiles had lower EC than the wealthiest quantiles.

EC was calculated for specific interventions and for a combination of interventions as an aggregate measure to highlight health system performance. There was a remarkable variability in EC estimates for different MCH services. Moreover, a substantial gap between CC and EC estimates was shown. This gap indicates that although women and children had adequate contact with health care providers through enhanced access to care, insufficient provision of important interventions limited the levels of care provided, as has been revealed in the high proportion of women and children not receiving good quality of care. CC of different interventions was much higher when compared to EC as quality lagged coverage and hence undermined effective delivery of services. CC is a widely used indicator that takes into account the use of services and the population with a need, missing an important component indicating health gain, i.e. quality (Ng *et al.*, 2014). Considering health care utilization or raw coverage measures solely can give misleading information regarding service delivery. Where quality of care is not considered, the results might be optimistic. For instance, high coverage of facility delivery or a pregnant woman visiting a health care provider does not necessarily mean women are getting a high standard of care (Austin *et al.*, 2014). Hence, applying measurement techniques that link health care use to the quality of service provided reveals a more accurate picture of the health care received by women and children. It is also important to consider that in the study by Martinez, the quality measurement strategy used might contribute to the overestimated EC value noted (Martínez *et al.*, 2011). Failure to choose the right indicators or to incorporate the multiple domains of measurement when assessing the quality of care in MCH may result in overestimated or underestimated EC values, eventually leading to wrong implications. Some of the included studies intended to calculate the EC of MCH interventions but reported only the CC measures due to difficulties in measuring the quality component.

The most challenging, but very important aspect of EC estimation, is measuring the quality component. The studies in this review measured quality through structural, process of care and outcome dimensions. The measurement technique used or the quality dimension evaluated determines the value of the EC estimate. Structural measures of quality typically include the characteristics of the resources in the health care system, including the availability of medicines, equipment and professionals. They provide a judgement on whether care is being provided under conditions that are either conducive or unfavourable to the provision of good care (Quentin *et al.*, 2019). In this review, facility capacity and readiness to provide services that contain domains such as knowledge, availability of services, human resources and basic commodities were used as structural attributes. Some authors argued that such structural measures might provide incomplete insight on the quality of care provided despite being relatively concrete and easy to measure (Rademakers *et al.*, 2011).

Process of care indicates all the actions that make up health care or what a provider delivers to maintain health either for healthy people or those diagnosed with a disease. Hence, this reflects how the institution is meeting generally accepted standards of practice. Process measures are predominantly used by researchers and provide an objective measurement of quality (Lilford *et al.*, 2007). The bulk of the studies included in our review used processes of care to measure quality. Few of the included articles reported research that used outcome metrics such as risk-adjusted mortality as a quality metric. Outcome measures demonstrate the end results of services and reflect all of the effects of health care on the health status of people, including changes to health status, behaviour, satisfaction and quality of life. They are important indicators of quality as the primary goal of health care is improving health status. However, the measurements of outcomes are difficult to obtain and may take considerable time (Hanefeld *et al.*, 2017). Some of the papers in this review described the use of scores and indexes concerning structures that were combined with indicators relating to the process of care, providing a more comprehensive view of the quality of care. Studies indicate that the recognition of the multifaceted nature of the quality of care is critical (Hanefeld *et al.*, 2017; Sixma *et al.*, 1998). However, most measurement approaches used often fail to address the complexities involved in understanding the quality of care. Biomarker information was also used to assess the quality of care. Evidence shows that biomarker data may help evaluate the quality of vaccines and immunization programmes, thereby providing improved insights into public health problems (Boerma *et al.*, 2001). Overall, the existence of multiple indicators to measure the quality of care may be related to the different processes involved in the development of protocols and guidelines for MCH services across countries. If the quality of interventions is not measured appropriately, coverage estimates may be overestimated or fail to show the true health gain by population.

Health care quality is multidimensional, and multiple indicators are used to measure constructs such as structure, process and outcome. The selection of constituting indicators and weights could influence the results of quality (Hanefeld *et al.*, 2017). To compare the gaps between CC and EC using the three quality domains, we considered articles that reported both CC and EC estimates. Studies examining family planning, ANC and post-partum care relied only on the process of care domain to measure quality. For each service, there were variations in the process indicators used across studies. Rwanda (Leslie *et al.*, 2017), Uganda (Leslie *et al.*, 2017) and Ethiopia (Marchant *et al.*, 2015) showed the narrowest gap between CC and EC for ANC, family planning and post-partum care, respectively. This may be due to both the low service coverage and shortfall in quality in these countries. EC is a construct of both coverage and quality; lower levels of both may result in lower gaps between CC and EC. Regarding delivery care, studies that used a combination of process and structure domains as a measure of quality (Larson *et al.*, 2017; Nesbitt *et al.*, 2013) exhibited higher gaps between CC and EC than those that used the structural domain only (Wang *et al.*, 2019; Willey *et al.*, 2018). This indicates that not considering the multiple domains of quality may underestimate the gap between EC and CC. A study in Burkina Faso (Koulidiati *et al.*, 2018) that used combined domains of process and structure to measure the quality of childcare seeking showed the highest gap between CC and EC as compared to a study that assessed eight countries separately using the process of care domain only (Leslie *et al.*, 2017). Comprehensive measures of quality that involve multiple dimensions or undergo a detailed assessment of sets of numerous indicators may reveal higher gaps between EC and CC. There appears to be great heterogeneity in the way in which quality and the indicators that construct its three domains are measured, limiting comparability across studies and countries.

Increasing attention is being paid by research to the examination of socio-economic inequality in MCH domains. So far, most of the work regarding inequities in health service delivery has been about access to care (World Health Organization, 2015). Wealth status, education and area of residence have a significant impact on access to and uptake of services. Studies show that the economically disadvantaged sections of society are the ones that face the greatest health problems (Ahmed *et al.*, 2010). Pro-rich inequalities for MCH coverage indicators are common (Victora *et al.*, 2016). Estimates of socio-economic inequalities using CC measures generally underestimate the level of inequalities compared with EC measures (Arsenault *et al.*, 2018). In Rwanda, as the EC increases, the socio-economic inequalities were widening mainly due to widening inequalities in quality, which calls for the importance of equitable quality improvement (Hategeka *et al.*, 2020). When efforts to increase coverage and quality are poorly monitored, an unbalanced benefit to the wealthier quintiles at the expense of the poorer quintiles may occur (Victora *et al.*, 2000; 2012). Evidence from other studies has noted a quality deficit in health services available to low-income groups (Sharma *et al.*, 2017; Barber *et al.*, 2007). The improvements in equity in the overall EC of MCH services observed in Kenya may possibly be linked to a range of interventions implemented over the study period (Mutua *et al.*, 2011; Noor *et al.*, 2007). Quality improvement can be a powerful tool for achieving equitable high-quality health care (Hirschhorn *et al.*, 2021). Previous evidence found that socio-economic inequities remain in most low-income countries when considering EC (Anindya *et al.*, 2021). Some of our reviewed articles indicated that the EC of MCH services tended to be inequitably distributed favouring those from the better-off socio-economic groups. The poorest quintiles had lower EC levels of services than the wealthiest quintiles. This can be explained by the fact that low-income women and children are less likely to receive high-quality care than their counterparts. Hence, as the quality of care is reduced, so does the EC. Emerging data highlight that predominantly, low-income people tend to be less informed about the quality of care and mostly live in rural areas and regions with poorly functioning health systems, where there is limited availability of quality facilities nearby and skilled professionals (Hardie and Landale, 2013). Other studies revealed that discriminatory treatment from health workers, cost of medical procedures, women’s empowerment and degree of patient activation to seek high-quality care appear to prevent women from low-income households to access high-quality services (McMahon *et al.*, 2014; Shiferaw *et al.*, 2013). Health system factors such as the disproportionate availability of services could also explain why low-income women get worse care (Bintabara *et al.*, 2019). The evidence we reviewed showed that the wealthiest women were usually more likely to report good quality ANC, delivery care, family planning and child health services such as immunization than the low-income women. The general trend in the literature is that maternal health indicators are particularly prone to such inequalities, with the rich–poor ratio reaching over 4-fold in some countries (Barros *et al.*, 2012; Boerma *et al.*, 2018). Prior research has documented pro-rich distribution of quality-adjusted coverage of MCH services (Sharma *et al.*, 2017; Rani *et al.*, 2008). In countries with adequate coverage of services, the richest quintiles are privileged to attain greater coverage than the poorest quintiles because they pick up the services first. According to Victora *et al.* (2012) and Victora *et al.* (2018) when the national coverage is very low, the services may not be available in both wealthy and least wealthy groups, resulting in lower levels of inequality. Some indicators and disease episodes tend to be higher among low-income people, and there tend to be more than 20% of the mothers and children in the lowest quintile of household wealth and fewer than 20% in the richest quintile. Such indicators that are often based on a much larger sample in the poorest than in the wealthiest quintile may reveal a pro-poor distribution. Consistent with the finding in Kenya (Nguhiu *et al.*, 2017), pro-poor inequality in the coverage of MCH services has been reported by other studies (Neal *et al.*, 2015; Joseph *et al.*, 2018).

Overall, it is notable that the gap between EC and CC is due to the poor quality of care at MCH services. This implies the need to devote greater attention to improving quality, along with expanding coverage. It is also important to prioritize improvement efforts to target vulnerable groups, first, including low-income people, since they have the worst quality of care and health outcomes. The low EC of MCH interventions or programmes highlighted in the review have implications for designing improved approaches that will contribute to achieving high EC. Previous studies have documented strategies such as the pathway approach to high EC, which facilitates systems thinking to better understand EC and health impacts. The Pathway approach consists of six key components: national readiness, system structures, management capacity, implementation strength, EC and impact. The first two components are applicable at a national level, whereas the third and the fourth pertain to the subnational level and the intervention delivery point, respectively. Various countries have used the pathway approach to guide strategic planning, monitoring and evaluation (Vaz *et al.*, 2020). It is increasingly recognized that health systems need to focus on achieving quality care for health gains in populations. Where there are better levels of access to MCH care, quality becomes even more evident as a critical factor for improved health outcomes (Kruk *et al.*, 2017). High-quality care should be the core of the health system (Busse *et al.*, 2019). A recent review of the evidence highlighted several approaches, which sought to improve the quality of maternal and childcare services. Among them were health promotion activities, health facility renovations and improvements, health provider training, incentives for service users, provision of outreach services, development of maternal and newborn health action plans, reviews of national clinical policies and standards and the formation of public–private partnerships at regional and national levels. These approaches targeted the patient, health care provider, organizational and the broader health system levels (Wilson *et al.*, 2020). Based on past frameworks like Donabedian’s, the authors proposed a new conceptual framework for high-quality health systems (Kruk *et al.*, 2018). The framework focused on three key domains: foundations, processes of care and quality impacts. Quality impacts include better health, confidence in system and economic impact. The process of care includes competent care and positive user experience, which are complementary elements necessary for achieving high-quality care. Foundations refer to the facilities and people required for care and include population, governance, platforms, workforce and tools. The authors argued that processes and outcomes provide a better measure of quality of health systems than structures. With regard to improvement strategies, governing for quality, redesigning service delivery, transforming the health workforce and raising demand for quality are key elements to achieving the highest quality of care.

The WHO established a framework that outlines the core components of quality services for the mother and the infant as one of the initiatives aimed at enhancing the quality of maternal and neonatal care. The framework identifies two key dimensions of quality: ‘provision of care’, including evidence-based practices, efficient information and referral systems, and ‘experience of care’, including effective communication, respect, dignity and emotional support. The cross-cutting areas of the framework include the availability of competent, motivated human resources and of the physical resources; identified are prerequisites for good quality of care in health facilities (Tunçalp *et al.*, 2015). In 2016, based on this framework, a list of WHO ‘Standards for improving maternal and newborn care in health facilities’ was released (World Health Organization, 2016).

Among the other approaches for promoting the uptake of quality care, interventions have been designed to support health financing on the demand and supply sides. One example is the performance-based financing programme on MCH services (James *et al.*, 2020). Evidence showed a favourable impact of Results-Based Financing (RBF) on structural, process and outcome quality indicators in low- and middle-income countries (Andrew, 2020; Friedman *et al.*, 2016; Zeng *et al.*, 2018b; 2018a; Tawfiq *et al.*, 2019; De Brouwere *et al.*, 2010). Malawi introduced the RBF to enhance obstetric care provision at emergency obstetric care facilities. This health system reform improved the EC of maternal and neonatal health by improving quality and allowing a large group of women to receive more effective care (Brenner *et al.*, 2018). Securing equitable access to quality health services is a key to realizing the benefits of quality health care. Countries like Mexico implemented important maternal health care policies such as ‘Prospera’ (formerly ‘Progresa’ or ‘Oportunidades’) and ‘Seguro Popular de Salud’. These policies aimed to improve the provision and quality of basic social services, including reproductive health, ultimately contributing to reducing gaps in EC. Positive synergies between these two policies in transforming public health efforts into greater EC and better MCH outcomes have been assessed. These successful initiatives specifically targeted the economically and socially disadvantaged population (Serván-Mori *et al.*, 2019).

Although there is a substantial body of research on the EC of MCH services, the literature is varied and scattered. This is the first systematic review to examine the EC of MCH services, the gaps between CC and EC, quality measuring methodologies and the distribution of EC across wealth quintiles. Our research adds to the current literature by demonstrating the disparity between crude and EC and the reliability of EC in determining actual service coverage while taking quality into account. Our review also revealed how rigorous and extensive analyses of quality indicators, including the specific components of quality domains, can lead to significant differences between CC and EC. Moreover, this study has pointed out inequalities in the EC of MCH services, favouring wealthy groups. No studies were located in high-income countries which might be because access to quality MCH services is well established in high-income countries making it a less area of concern.

## Conclusion

MCH services need to ensure a combined focus on both accessibility of services and the provision of high-quality care for better outcomes. The results of the review underscore the importance of looking beyond raw coverage measures or health care utilization indicators. Comparing crude and quality-adjusted coverage of MCH interventions portrayed the existing gap between these estimates. In addition, the findings demonstrate that the effectiveness of MCH interventions is low. Ineffective care indicates missed opportunities to achieve better outcomes, and such findings underpin the importance of prioritizing quality of care alongside efforts to improve MCH problems. The results also suggest that better measurement, as well as greater consensus on the metrics of quality of services, will be needed to ensure appropriate monitoring and evaluation of health system performance. Hence, EC estimates adjusted with appropriate quality measures provide a powerful mechanism for revealing gaps in the delivery of effective care. This, in turn, contributes to the efforts to overcome the burden of maternal and newborn death. Also, highlighted by our results, EC remains disproportionately lower among low-income groups. This necessitates the call for evidence-based approaches to reduce inequalities in MCH service provision and quality. Generally, as the global community turns its attention towards Universal Health Coverage, it will be crucial to improve quality, close the gap between CC and EC, use standard quality measurement strategies and ensure equitable distribution across all levels of care.

## Supplementary Material

czac034_Supp

## Reflexivity Statement

The author team represents diverse professional backgrounds with a range of research experience and expertise. Each author had a unique contribution that was incorporated into the review process. The author team represents researchers from high-income countries (AH and AD) and low- and middle-income countries (AF). Two authors are female and one author is male. This study contributes to AF’s PhD study through the University of Technology Sydney. AF was supported by the University of Technology Sydney to develop and refine writing skills, including through the facilitation of scientific writing workshops. AH and AD are professors of public health and experienced researchers.
