# Supplementary material for: Evaluations of effective coverage of maternal and child health services: A systematic review
Source: Health Policy Plan. 2022 Apr 23;37(7):895–914. doi: 10.1093/heapol/czac034 (PMC9347022; doi:10.1093/heapol/czac034)
Supplement: czac034_Supp [file czac034_supp.zip › Supplementary_File_1__Search_strategy.docx]

| **Databases** | **Search Terms** |
| --- | --- |
| **Web of Science** | ts=(mother* OR female OR maternal OR women OR children OR child OR infant OR pediatric* OR paediatric* OR newborn OR neonate OR childhood ) **AND**  ts=("Maternal and child health services" OR “maternal & child health services” OR "Maternal and child health service" OR "Maternal-Child Health Services" OR "Maternal-Child Health Service" OR "Maternal Health Services" OR "Maternal Health Service" OR "Maternal Health" OR "Child Health Services" OR "Child Health Service" OR "Child health" OR “child health care” OR "Family Planning Services" OR "Family Planning Service" OR "Prenatal Care" OR pregnancy OR "perinatal care" OR "Obstetric Delivery" OR "Infant care" OR "Postnatal Care" OR "Health Services" OR “health service” OR "Maternal-Child Health" OR "obstetric care" OR "Delivery care" OR delivery OR "Newborn care" OR "Women's health" OR "Women's Health Service" OR "Women's Health Services" OR "Infant health" OR fever OR "Respiratory Tract Infections" OR diarrhoea OR diarrhea OR "Curative child health services" OR "Curative child health service" OR "Neonatal care " OR ” neonatal health” OR "Child treatment" OR "Family planning" OR "Antenatal care" OR "Facility delivery" OR "Skilled birth attendance" OR "skilled attendance at birth" OR "skilled attendance" OR "Institutional delivery" OR "Institutional deliveries" OR "Postpartum care" OR immunization OR vaccination OR "Newborn health" OR "Management of child illness" OR "Management of child illnesses" OR "childhood illness management" OR “childhood disease” OR “childhood illnesses” OR "Management of childhood illnesses" OR "maternal and neonatal health" OR "Delivery of Health Care" OR “health care” OR “health facility” OR "Maternal health care services" OR "Maternal and newborn health*" OR “maternal and child health*” OR “reproductive health service*” OR “reproductive health” ) **AND** ts=("Effective coverage " OR "Crude coverage" OR "Quality-adjusted coverage" OR "Quality adjusted coverage" OR "Quality contacts" OR “high quality contacts” OR "quality-adjusted contacts" ) |
| **Medline** | exp Mothers/ OR Mother.mp. OR Female/ OR maternal.mp. OR exp Women/ OR Children.mp. OR exp Child/ OR exp Child, Preschool/ OR preschool child.mp. OR exp Adolescent/ OR exp Adult/ OR exp Young Adult/ OR exp Middle Aged/ OR exp Infant/ OR exp Infant, Newborn/ OR exp Pediatrics/ OR Newborn.mp. OR childhood.mp. OR exp Adult Children/ OR Neonate.mp. **AND** "Maternal and child health services".mp. OR "Maternal and child health service".mp. OR "Maternal & child health services".mp. OR "Maternal and child health".mp. OR exp Maternal-Child Health Services/ OR exp Maternal Welfare/ OR exp Maternal Health Services/ OR Maternal health service.mp. OR exp Maternal Health/ OR Maternal health services.mp. OR exp Child Health Services/ OR Child health service.mp. OR Child health services.mp. OR exp Child Health/ OR Child health care.mp. OR Maternal-Child Health.mp. OR Maternal care.mp. OR exp Prenatal Care/ OR exp Pregnancy/ OR exp Perinatal Care/ OR exp Delivery, Obstetric/ OR exp Infant Care/ OR exp Postnatal Care/ OR post-natal care.mp. OR exp Family Planning Services/ OR exp Pregnancy Complications/ OR Obstetric delivery.mp. OR obstetric care.mp. OR delivery care.mp. OR Newborn care.mp. OR exp Women's Health/ OR exp Women's Health Services/ OR exp Infant Health/ OR Neonatal care.mp. OR exp Fever/ OR exp Respiratory Tract Infections/ OR Respiratory tract infection.mp. OR exp Diarrhea/ OR Curative child health services.mp. OR child treatment.mp. OR Childhood disease.mp. OR Sick child visits.mp. OR Management of child illness.mp. OR Management of child illnesses.mp. OR childhood illness management.mp. OR Family planning.mp. OR Antenatal care.mp. OR Facility delivery.mp. OR Skilled birth attendance.mp. OR skilled attendance.mp. OR Institutional delivery.mp. OR institutional deliveries.mp. OR Postpartum care.mp. OR *Immunization/ OR *Vaccination/ OR Newborn health.mp. OR "Maternal and newborn health".mp. OR Maternal-Child Care.mp. OR "maternal and neonatal health ".mp. OR Neonatal health.mp. OR exp "Health Services Needs and Demand"/ OR exp Health Services Research/ OR exp Health Services Accessibility/ OR "Delivery of Health Care"/ OR Health care delivery.mp. OR health care access.mp. OR exp Health Services/ OR exp "Patient Acceptance of Health Care"/ OR exp Reproductive Health Services/ OR exp Reproductive Health/ OR Child health care.mp. OR child birth.mp. OR health care facility.mp. OR maternal health care services.mp. OR healthcare.mp. OR health interventions.mp. **AND** effective coverage.mp. OR Crude coverage.mp. OR quality-adjusted coverage.mp. OR Quality contacts.mp. OR quality-adjusted contacts.mp. OR High quality contacts.mp. |
| **Embase** | Mothers.mp. OR exp mother/ OR exp female/ OR maternal.mp. OR women.mp. OR Children.mp. OR exp child/ OR exp preschool child/ OR exp adolescent/ OR exp adult/ OR exp young adult/ OR exp middle aged/ OR exp infant/ OR exp pediatrics/ OR exp newborn/ OR exp childhood/ OR exp adult child/ OR Neonate.mp.  **AND** "Maternal and child health services".mp. OR Maternal & child health services.mp. OR "Maternal and child health service".mp. OR exp maternal child health care/ OR "Maternal and child health".mp. OR "Maternal-Child Health Services".mp. OR exp maternal welfare/ OR Maternal Health Services.mp. OR exp maternal health service/ OR Maternal Health.mp. OR Child Health Services.mp. OR Child health service.mp. OR exp child health/ OR exp child health care/ OR Maternal-Child Health.mp. OR exp maternal care/ OR "Maternal and newborn health".mp OR Maternal-Child Care.mp. OR "maternal and neonatal health".mp. OR exp prenatal care/ OR exp pregnancy/ OR exp perinatal care/ OR exp infant care/ OR Infant health.mp. OR Neonatal care.mp. OR Neonatal health.mp. OR exp postnatal care/ OR post-natal care.mp. OR Family Planning Services.mp. OR exp pregnancy complication/ OR exp obstetric delivery/ OR obstetric care.mp. OR delivery care.mp. OR exp newborn care/ OR Newborn health.mp. OR exp women's health/ OR Women's Health Services.mp. OR exp fever/ OR exp respiratory tract infection/ OR exp diarrhea/ OR Curative child health services.mp. OR child treatment.mp. OR exp childhood disease/ OR Sick child visits.mp. OR Management of child illness.mp. OR Management of childhood illnesses.mp. OR Management of childhood illnesses.mp. OR exp family planning/ OR Antenatal care.mp. OR Facility delivery.mp. OR Skilled birth attendance.mp. OR skilled attendance.mp. OR Institutional delivery.mp. OR institutional deliveries.mp. OR Postpartum care.mp. OR exp immunization/ OR exp vaccination/ OR exp health services research/ OR exp health care need/ OR Health Services Accessibility.mp. OR exp health care delivery/ OR Delivery of Health Care.mp. OR exp health care access/ OR exp health service/ OR Health Services.mp OR Reproductive health services.mp. OR exp child health care/ OR child health care/ OR exp childbirth/ OR exp health care facility/ OR maternal health care services.mp OR exp health care/ OR health interventions.mp. **AND** "Effective coverage ".mp. OR Crude coverage.mp. OR Quality-adjusted coverage.mp. OR Quality adjusted coverage.mp. OR Quality contacts.mp. OR High quality contacts.mp. |
| **Maternity & Infant Care** | Mothers.de. OR Mother.mp. OR Female.de. OR Maternal.de. or maternal.mp. OR Women.de. or women.mp. OR Child.de. or Children.mp. OR Child - preschool.de. OR Adolescent.de. OR Adult.de. OR Middle aged.de. OR Infant.mp. or Infant.de. OR Infant - newborn.de. OR Paediatrics.de. OR newborn.de. or Newborn.mp. OR Childhood.de. or childhood.mp. OR Neonate.mp. or Neonate.de. **AND** “Maternal & child health services".mp. OR "Maternal & child health service".mp. OR "Maternal and child health services".mp. OR "maternal child health care".mp. OR "Maternal and child health".mp. OR "Maternal-Child Health Services".mp. OR Maternal health services.de. OR "Maternal health service".mp. OR "Maternal Health Services".mp. OR Maternal health.de. OR Child health services.de. OR "Child health service".mp. OR "Child health services".mp. OR Child health.de. OR "Child health care".mp. OR "Maternal-Child Health".mp. OR Maternal care.de. OR "Maternal-Child Care".mp. OR (maternal and neonatal health).mp. [mp=abstract, heading word, title] OR Prenatal care.de. OR Pregnancy.de. OR Perinatal care.de. OR Delivery.de. OR Infant care.de. OR Infant health.de. OR Neonatal care.de. OR "Neonatal health".mp. OR "Postnatal Care".mp. or Postnatal care.de. OR Family planning services.de. or "Family Planning Services".mp. OR Pregnancy complications.de. OR "Obstetric delivery".mp. OR "obstetric care".mp. OR "delivery care".mp. OR "Newborn care".mp. OR "Newborn health".mp. OR Women's health.de. OR Women's health services.de. OR Fever.de. OR Respiratory tract infections.de. OR Respiratory tract infection.mp. OR Diarrhoea.de. OR "child treatment".mp. OR "Childhood disease".mp. OR Family planning.de. or "Family planning".mp. OR Antenatal care.de. or "Antenatal care".mp. OR "Facility delivery".mp. OR Skilled birth attendance.mp. [mp=abstract, heading word, title] OR skilled attendance.mp. [mp=abstract, heading word, title] OR Institutional delivery.mp. [mp=abstract, heading word, title] OR Postpartum care.de. or Postpartum care.mp. OR Immunization.mp. or Immunization.de. OR Health services research.de. OR health care need.mp. OR "Health services needs and demands".de. OR Health services accessibility.de. OR "Delivery of health care".de. OR Health care delivery.mp. OR health care access.mp. or Access to health care.de. OR Health services.de. or health service.mp. OR health Services.mp. OR "Patient acceptance of health care".de. OR Reproductive health services.de. OR Reproductive health services.mp. OR Reproductive health.de. OR Reproductive health.mp. OR Child health care.mp. OR health care facility.mp. or Health facility environment.de. OR Health facilities.de. or Health facility.mp. OR maternal health care services.mp. OR healthcare.mp. OR health interventions.mp. **AND** Effective coverage.mp. [mp=abstract, heading word, title] OR Crude coverage.mp. [mp=abstract, heading word, title] |
| **CINHAL** | (MH "Mothers+") OR "Mother" OR (MH "Female") OR "maternal" OR (MH "Women+") OR "Children" OR (MH "Child+") OR (MH "Child, Preschool") OR (MH "Adult+") OR (MH "Young Adult") OR (MH "Middle Age") OR (MH "Infant+") OR (MH "Infant, Newborn+") OR (MH "Pediatrics+") OR "Newborn" OR "childhood" OR (MH "Adult Children") OR "Neonate" **AND** "“Maternal & child health services”" OR ""Maternal and child health services"" OR ""maternal child health care"" OR ""Maternal and child health"" OR ""Maternal-Child Health Services"" OR (MH "Maternal Welfare") OR (MH "Maternal Health Services+") OR ""Maternal health service"" OR ""Maternal Health"" OR (MH "Child Health Services+") OR ""Child health service"" OR (MH "Child Health") OR ""Child health care"" OR (MH "Maternal-Child Health") OR ""Maternal care"" OR ""Maternal and newborn health"" OR (MH "Maternal-Child Care") OR ""maternal and neonatal health"" OR (MH "Prenatal Care") OR (MH "Pregnancy+") OR (MH "Perinatal Care") OR (MH "Delivery, Obstetric+") OR (MH "Infant Care+") OR ""Infant health"" OR ""Neonatal care"" OR ""Neonatal health"" OR (MH "Postnatal Care+") OR ""post-natal care"" OR ""Family Planning Services"" OR (MH "Pregnancy Complications+") OR ""Obstetric delivery"" OR (MH "Obstetric Care+") OR ""delivery care"" OR ""Newborn care"" OR ""Newborn health"" OR (MH "Women's Health") OR (MH "Women's Health Services") OR (MH "Fever+") OR (MH "Respiratory Tract Infections") OR (MH "Diarrhea") OR "Diarrhoea" OR "Diarrhea" OR ""Curative child health services"" OR ""child treatment"" OR ""Childhood disease"" OR ""Childhood illnesses"" OR ""Sick child visits"" OR ""Management of child illness"" OR ""Management of childhood illnesses"" OR ""childhood illness management"" OR (MH "Family Planning+") OR ""Family planning"" OR ""Antenatal care"" OR ""Facility delivery"" OR ""Skilled birth attendance"" OR ""skilled attendance"" OR ""Institutional delivery"" OR ""institutional deliveries"" OR ""Postpartum care"" OR (MH "Immunization+") OR "Immunization" OR "vaccination" OR (MH "Health Services Needs and Demand+") OR (MH "Health Services Research+") OR ""health care need"" OR (MH "Health Services Accessibility+") OR (MH "Health Care Delivery+") OR ""health care access"" OR ""health service"" OR (MH "Health Services+") OR "services" OR ""Reproductive health services"" OR (MH "Reproductive Health") OR ""Child health care"" OR ""Reproductive health"" OR (MH "Childbirth+") OR ""health care facility"" OR "Childbirth" OR ""Health facility"" OR (MH "Health Facilities+") OR ""maternal health care services"" OR "healthcare" OR ""health interventions"" **AND** ""Effective coverage"" OR ""Crude coverage"" OR ""Quality-adjusted coverage"" OR ""Quality adjusted coverage"" OR ""quality-adjusted contacts"" OR ""High quality contacts"" |
| **Scopus** | ( TITLE-ABS-KEY ( mother* OR female OR maternal OR wom?n OR children OR child OR infant OR pediatric* OR paediatric* OR newborn OR neonate OR childhood ) ) AND ( TITLE-ABS-KEY ( "Maternal and child health services" OR “maternal & child health services” OR "Maternal and child health service" OR "Maternal-Child Health Services" OR "Maternal-Child Health Service" OR "Maternal Health Services" OR "Maternal Health Service" OR "Maternal Health" OR "Child Health Services" OR "Child Health Service" OR "Child health" OR “child health care” OR "Family Planning Services" OR "Family Planning Service" OR "Prenatal Care" OR pregnancy OR "perinatal care" OR "Obstetric Delivery" OR "Infant care" OR "Postnatal Care" OR "Health Services" OR “health service” OR "Maternal-Child Health" OR "obstetric care" OR "Delivery care" OR delivery OR "Newborn care" OR "Wom?n's health" OR "Wom?n's Health Service" OR "Wom?n's Health Services" OR "Infant health" OR fever OR "Respiratory Tract Infections" OR diarrhoea OR diarrhea OR "Curative child health services" OR "Curative child health service" OR "Neonatal care " OR ” neonatal health” OR "Child treatment" OR "Family planning" OR "Antenatal care" OR "Facility delivery" OR "Skilled birth attendance" OR "skilled attendance at birth" OR "skilled attendance" OR "Institutional delivery" OR "Institutional deliveries" OR "Postpartum care" OR immunization OR vaccination OR "Newborn health" OR "Management of child illness" OR "Management of child illnesses" OR "childhood illness management" OR “childhood disease” OR “childhood illnesses” OR "Management of childhood illnesses" OR "maternal and neonatal health" OR "Delivery of Health Care" OR “health care” OR “health facility” OR "Maternal health care services" OR "Maternal and newborn health*" OR “maternal and child health*” OR “reproductive health service*” OR “reproductive health” ) ) **AND** ( TITLE-ABS-KEY ( "Effective coverage " OR "Crude coverage" OR "Quality-adjusted coverage" OR "Quality adjusted coverage" OR "Quality contacts" OR “high quality contacts” OR "quality-adjusted contacts" ) ) |
| **Hand search** | Varieties of key terms from the above-mentioned were used. |
