# Supplementary material for: Evaluations of effective coverage of maternal and child health services: A systematic review
Source: Health Policy Plan. 2022 Apr 23;37(7):895–914. doi: 10.1093/heapol/czac034 (PMC9347022; doi:10.1093/heapol/czac034)
Supplement: czac034_Supp [file czac034_supp.zip › Supplementary_File_2_Quality_assesment.docx]

Table S2: Quality assessment results of the included studies using Joanna Briggs Institute’s standardized critical appraisal instrument for prevalence studies

| Citation | Q1 | Q2 | Q3 | Q4 | Q5 | Q6 | Q7 | Q8 | Q9 |
| --- | --- | --- | --- | --- | --- | --- | --- | --- | --- |
| Hategeka C, *et al*., 2020 [[1](#_ENREF_1)] | Y | Y | Y | Y | Y | N | Y | Y | Y |
| Nguhiu et al., 2017 [[2](#_ENREF_2)] | Y | Y | Y | Y | Y | Y | Y | Y | Y |
| Joseph et al., 2020 [[3](#_ENREF_3)] | Y | Y | Y | Y | Y | Y | Y | Y | Y |
| Lozano et al., 2006 [[4](#_ENREF_4)] | Y | Y | Y | Y | N | N | Y | N | Y |
| Yakob et al., 2019 [[5](#_ENREF_5)] | Y | Y | Y | Y | Y | Y | Y | Y | Y |
| Gutiérrez, J. P. et al., 2013. [[6](#_ENREF_6)] | Y | Y | Y | Y | U | N | Y | N | Y |
| Martínez S. et al., 2011 [[7](#_ENREF_7)] | Y | Y | Y | Y | U | N | U | N | Y |
| Wang et al., 2019 [[8](#_ENREF_8)] | Y | Y | Y | Y | Y | Y | Y | Y | Y |
| Nesbitt et al., 2013 [[9](#_ENREF_9)] | Y | Y | Y | Y | Y | Y | Y | Y | Y |
| Koulidiati J-l, et al. , 2018 [[10](#_ENREF_10)] | N | N | Y | Y | N | Y | Y | Y | Y |
| Leslie HH, et al., 2017 [[11](#_ENREF_11)] | Y | Y | Y | Y | Y | Y | Y | Y | Y |
| Carter et al., 2018 [[12](#_ENREF_12)] | Y | Y | Y | Y | Y | Y | Y | Y | Y |
| Leslie et al., 2019 [[13](#_ENREF_13)] | Y | Y | N | Y | Y | Y | Y | Y | Y |
| Marchant et al., 2015 [[14](#_ENREF_14)] | Y | Y | Y | Y | Y | Y | Y | Y | Y |
| Okawa S. et al., 2019 [[15](#_ENREF_15)] | N | N | Y | Y | Y | Y | Y | Y | Y |
| Murphy et al., 2018 [[16](#_ENREF_16)] | Y | Y | Y | Y | Y | Y | Y | Y | Y |
| Willey et al., 2018 [[17](#_ENREF_17)] | Y | Y | Y | Y | Y | Y | Y | Y | Y |
| Larson et al., 2016 [[18](#_ENREF_18)] | Y | N | Y | Y | Y | Y | Y | Y | Y |
| Munos et al., 2018 [[19](#_ENREF_19)] | Y | Y | Y | Y | Y | Y | Y | Y | Y |
| Hodgins et al., 2014 [[20](#_ENREF_20)] | Y | Y | Y | Y | Y | Y | Y | Y | Y |
| Idzerda et al, 2011 [[21](#_ENREF_21)] | Y | Y | Y | Y | U | N | U | U | Y |
| Engle-Stone et al., 2015 [[22](#_ENREF_22)] | Y | Y | Y | Y | Y | Y | Y | Y | Y |
| Colson et al., 2015 [[23](#_ENREF_23)] | Y | Y | Y | Y | Y | Y | Y | Y | Y |
| Venkateswaran et al., 2019 [[24](#_ENREF_24)] | Y | Y | Y | Y | N | N | Y | Y | Y |
| Kyei et al., 2012 [[25](#_ENREF_25)] | Y | Y | Y | Y | Y | Y | Y | Y | Y |
| Travassos et al., 2016 [[26](#_ENREF_26)] | N | Y | Y | Y | N | Y | Y | Y | Y |
| Nguyen PH, et al., 2021 [[27](#_ENREF_27)] | Y | Y | Y | Y | N | N | Y | Y | Y |
| Legend  Y=Yes, N=No, U=Unclear | | | | | | | | | |

Q1: Was the sample frame appropriate to address the target population?

Q2: Were study participants sampled in an appropriate way?

Q3: Was the sample size adequate?

Q4: Were the study subjects and the setting described in detail?

Q5: Was the data analysis conducted with sufficient coverage of the identified sample?

Q6: Were valid methods used for the identification of the condition?

Q7: Was the condition measured in a standard, reliable way for all participants?

Q8: Was there appropriate statistical analysis?

Q9: Was the response rate adequate, and if not, was the low response rate managed appropriately?

**References**

1. Hategeka C, Arsenault C, Kruk ME: **Temporal trends in coverage, quality and equity of maternal and child health services in Rwanda, 2000–2015**. *BMJ global health* 2020, **5**(11):e002768.

2. Nguhiu PK, Barasa EW, Chuma J: **Determining the effective coverage of maternal and child health services in Kenya, using demographic and health survey data sets: tracking progress towards universal health coverage**. *Tropical Medicine & International Health* 2017, **22**(4):442-453.

3. Joseph NT, Piwoz E, Lee D, Malata A, Leslie HH, Group CCTW: **Examining coverage, content, and impact of maternal nutrition interventions: the case for quality-adjusted coverage measurement**. *Journal of global health* 2020, **10**(1).

4. Lozano R, Soliz P, Gakidou E, Abbott-Klafter J, Feehan DM, Vidal C, Ortiz JP, Murray CJ: **Benchmarking of performance of Mexican states with effective coverage**. *The Lancet* 2006, **368**(9548):1729-1741.

5. Yakob B, Gage A, Nigatu TG, Hurlburt S, Hagos S, Dinsa G, Bowser D, Berman P, Kruk ME, Tekle E: **Low effective coverage of family planning and antenatal care services in Ethiopia**. *International Journal for Quality in Health Care* 2019, **31**(10):725-732.

6. Gutiérrez JP: **Gaps in effective coverage by socioeconomic status and poverty condition**. *Salud publica de Mexico* 2013, **55**:S106-111.

7. Martínez S, Carrasquilla G, Guerrero R, Gómez-Dantés H, Castro V, Arreola-Ornelas H, Bedregal P: **Effective coverage of health interventions in Latin America and the Caribbean: metrics for the assessment of health systems performance**. *Salud publica de Mexico* 2011, **53**:s78-84.

8. Wang W, Mallick L, Allen C, Pullum T: **Effective coverage of facility delivery in Bangladesh, Haiti, Malawi, Nepal, Senegal, and Tanzania**. *PloS one* 2019, **14**(6):e0217853.

9. Nesbitt RC, Lohela TJ, Manu A, Vesel L, Okyere E, Edmond K, Owusu-Agyei S, Kirkwood BR, Gabrysch S: **Quality along the continuum: a health facility assessment of intrapartum and postnatal care in Ghana**. *PloS one* 2013, **8**(11):e81089.

10. Koulidiati J-L, Nesbitt RC, Ouedraogo N, Hien H, Robyn PJ, Compaoré P, Souares A, Brenner S: **Measuring effective coverage of curative child health services in rural Burkina Faso: a cross-sectional study**. *BMJ open* 2018, **8**(5):e020423.

11. Leslie HH, Ndiaye Y, Kruk ME: **Effective coverage of primary care services in eight high-mortality countries**. *BMJ Global Health* 2017, **2**(3).

12. Carter ED, Ndhlovu M, Eisele TP, Nkhama E, Katz J, Munos M: **Evaluation of methods for linking household and health care provider data to estimate effective coverage of management of child illness: results of a pilot study in Southern Province, Zambia**. *Journal of global health* 2018, **8**(1).

13. Leslie HH, Doubova SV, Pérez-Cuevas R: **Assessing health system performance: effective coverage at the Mexican Institute of social security**. *Health policy and planning* 2019, **34**(Supplement_2):ii67-ii76.

14. Marchant T, Tilley-Gyado RD, Tessema T, Singh K, Gautham M, Umar N, Berhanu D, Cousens S, Schellenberg JRA: **Adding content to contacts: measurement of high quality contacts for maternal and newborn health in Ethiopia, north east Nigeria, and Uttar Pradesh, India**. *PloS one* 2015, **10**(5):e0126840.

15. Okawa S, Win HH, Leslie HH, Nanishi K, Shibanuma A, Aye PP, Jimba M: **Quality gap in maternal and newborn healthcare: a cross-sectional study in Myanmar**. *BMJ global health* 2019, **4**(2):e001078.

16. Murphy GA, Gathara D, Mwachiro J, Abuya N, Aluvaala J, English M: **Effective coverage of essential inpatient care for small and sick newborns in a high mortality urban setting: a cross-sectional study in Nairobi City County, Kenya**. *BMC medicine* 2018, **16**(1):1-11.

17. Willey B, Waiswa P, Kajjo D, Munos M, Akuze J, Allen E, Marchant T: **Linking data sources for measurement of effective coverage in maternal and newborn health: what do we learn from individual-vs ecological-linking methods?** *Journal of global health* 2018, **8**(1).

18. Larson E, Vail D, Mbaruku GM, Mbatia R, Kruk ME: **Beyond utilization: measuring effective coverage of obstetric care along the quality cascade**. *International Journal for Quality in Health Care* 2017, **29**(1):104-110.

19. Munos MK, Maiga A, Do M, Sika GL, Carter ED, Mosso R, Dosso A, Leyton A, Khan SM: **Linking household survey and health facility data for effective coverage measures: a comparison of ecological and individual linking methods using the Multiple Indicator Cluster Survey in Côte d’Ivoire**. *Journal of global health* 2018, **8**(2).

20. Hodgins S, D'Agostino A: **The quality–coverage gap in antenatal care: toward better measurement of effective coverage**. *Global Health: Science and Practice* 2014, **2**(2):173-181.

21. Idzerda L, Adams O, Patrick J, Schrecker T, Tugwell P: **Access to primary healthcare services for the Roma population in Serbia: a secondary data analysis**. *BMC international health and human rights* 2011, **11**(1):1-14.

22. Engle-Stone R, Nankap M, Ndjebayi AO, Vosti SA, Brown KH: **Estimating the effective coverage of programs to control vitamin A deficiency and its consequences among women and young children in Cameroon**. *Food and nutrition bulletin* 2015, **36**(3_suppl):S149-S171.

23. Colson KE, Zúñiga-Brenes P, Ríos-Zertuche D, Conde-Glez CJ, Gagnier MC, Palmisano E, Ranganathan D, Usmanova G, Salvatierra B, Nazar A: **Comparative estimates of crude and effective coverage of measles immunization in low-resource settings: findings from Salud Mesoamérica 2015**. *PloS one* 2015, **10**(7):e0130697.

24. Venkateswaran M, Bogale B, Abu Khader K, Awwad T, Friberg IK, Ghanem B, Hijaz T, Mørkrid K, Frøen JF: **Effective coverage of essential antenatal care interventions: a cross-sectional study of public primary healthcare clinics in the West Bank**. *PloS one* 2019, **14**(2):e0212635.

25. Kyei NN, Chansa C, Gabrysch S: **Quality of antenatal care in Zambia: a national assessment**. *BMC pregnancy and childbirth* 2012, **12**(1):1-11.

26. Travassos MA, Beyene B, Adam Z, Campbell JD, Mulholland N, Diarra SS, Kassa T, Oot L, Sequeira J, Reymann M: **Immunization coverage surveys and linked biomarker serosurveys in three regions in Ethiopia**. *PLoS One* 2016, **11**(3):e0149970.

27. Nguyen PH, Khương LQ, Pramanik P, Billah SM, Menon P, Piwoz E, Leslie HH: **Effective coverage of nutrition interventions across the continuum of care in Bangladesh: insights from nationwide cross-sectional household and health facility surveys**. *BMJ open* 2021, **11**(1):e040109.
