# Supplementary material for: Evaluations of effective coverage of maternal and child health services: A systematic review
Source: Health Policy Plan. 2022 Apr 23;37(7):895–914. doi: 10.1093/heapol/czac034 (PMC9347022; doi:10.1093/heapol/czac034)
Supplement: czac034_Supp [file czac034_supp.zip › Supplementary_File_3.docx]

Figure S3-A. The effective coverage (EC), crude coverage (CC), and the gap between EC & CC of antenatal care (ANC) across the studies

Figure S3-B. The effective coverage (EC), crude coverage (CC), and the gap between EC & CC of family planning across the studies

Figure S3-C. The effective coverage (EC), crude coverage (CC), and the gap between EC & CC of skilled birth attendance (SBA) across the studies

Figure S3-D. The effective coverage (EC), crude coverage (CC), and the gap between EC & CC of postnatal care across the studies

Figure S3-E. The effective coverage (EC), crude coverage (CC), and the gap between EC & CC of care seeking for sick child across the studies
