# Supplementary material for: Evaluations of effective coverage of maternal and child health services: A systematic review
Source: Health Policy Plan. 2022 Apr 23;37(7):895–914. doi: 10.1093/heapol/czac034 (PMC9347022; doi:10.1093/heapol/czac034)
Supplement: czac034_Supp [file czac034_supp.zip › Supplementary_File_4_Quality_measurment_strategies.docx]

**Table S4. Quality measurement strategies of studies assessing the effective coverage of maternal and child health services**

| **Author** | **Domain** | **Quality indices** | |
| --- | --- | --- | --- |
| Hategeka C, *et al*., 2020  [[1](#_ENREF_1)] | Process | Antenatal care (ANC) | Blood pressure, urine and blood samples taken during ANC with skilled provider, iron supplementation, told about danger signs or where to go in case of complications during ANC with skilled provider (counselling) |
|  |  | Postpartum care | Postpartum check-up for mothers in a health facility after delivery and before discharge, women who were examined or asked questions about their health within one hour of delivery (Timely care), children who had all three doses of the DPT vaccine by one year of age |
|  |  | Care for sick children | Received antibiotics when seeking care at a facility for symptoms of pneumonia  Children who had blood taken from finger or heel for testing / tested for malaria (For fever)  Children who received oral rehydration therapy (from oral rehydration salts (ORS), pre-packaged ORS liquid or other homemade fluids)/ ORT |
| Nguhiu et al., 2017 [[2](#_ENREF_2)] | Process | Family planning | Facility level score based on the presence of client privacy during consultation, availability of reproductive health counselling visual aids and record tools, and reproductive health commodity management practices in a facility |
|  |  | ANC | Blood pressure taken, urine sample taken, blood sample taken, respondent informed about pregnancy complications, iron tablets/syrup prescribed, and a drug for intestinal parasites prescribed, during any ANC visit |
|  |  | Delivery care | Routine rooming in with the mother, routine weighing of new-borns, complete examination of new-borns before discharge, administration of BCG before discharge and other indicators. |
|  |  | Exclusive breastfeeding | Breastfeeding only, with no other complementary feed offered reported |
|  |  | Immunization | Observed or health worker reported availability of at least one working weighing scale and thermometer, and routinely performed processes including use of guidelines to assess and treat sick children, routine weighing, temperature taking and recording, assessment of immunization status and keeping of individual patient records. |
|  |  | Management of diarrhoea | Children who had diarrhoea in the preceding 4 weeks, who were given the guideline recommended oral rehydration salt mixture |
|  |  | Care seeking for acute respiratory infection (ARI) | Observed or health worker reported availability of at least one working weighing scale and thermometer, and routinely performed processes including use of guidelines to assess and treat sick children, routine weighing, temperature taking and recording, assessment of immunisation status and keeping of individual patient records |
|  |  | Use of ITN | Proportion of children and pregnant women who actually slept under an insecticide treated net in the preceding night |
| Nguyen PH, et al., 2021 [[3](#_ENREF_3)] | Structure | ANC | Human resources► Staff with any training on ANC Guidelines, national guidelines for ANC, visual aids for client education.  Basic equipment ► Adult weighing scale, tape measure for fundal height, Blood pressure apparatus, Stethoscope, Foetal stethoscope.  Diagnostic capacity. ► Haemoglobin. ► Urine protein.  Essential medicines. ► Iron tablets. ► IFA tablets. |
|  |  | Delivery care | Human resources ► Staff with any training on IMPACT  Guidelines ► Guidelines on basic birth care  BEmONC ► Guidelines on comprehensive birth care: CEmONC  Basic equipment ► Infant scale ► Manual or digital BP apparatus |
|  |  | Child growth monitoring | Human resources ► Staff with any training on growth monitoring  Guidelines ► Guidelines for growth monitoring  Basic equipment: ► Scale ► Length or height board ► Tape for measuring head ► Growth chart |
|  |  | Sick child care | Human resources ► Staff with any training on IMCI  Guidelines ► IMCI guideline: national guidelines for IMCI, IMCI chart booklet, IMCI card, other visual aids  Basic equipment ► Scale  Diagnostic capacity ► Haemoglobin  Essential medicines: ► ORS ► Albendazole/ mebendazole ► Iron tablet ► Vitamin A ► Zinc tablet/zinc sulphate syrup. |
| Larson et al., 2016 [[4](#_ENREF_4)] | Process & structure | Obstetric care | Facility infrastructure(toilet, electricity, water)  Availability of equipment, supplies and medicines (stainless steel bowls, stethoscope, uterotonic, magnesium sulphate, blood pressure cuff etc.)  Health worker knowledge and competence;  Provision of routine obstetric services; (baby breastfed within 1 hr, APGAR, HIV test, baby weighed, maternal blood pressure ,Partograph)  Provision of emergency obstetric and newborn services(uterotonic, removal of conception retained products, newborn resuscitation, antibiotics, anticonvulsants, manual removal of placenta) |
| Nesbitt et al., 2013 [[5](#_ENREF_5)] | Process & structure | Skilled birth attendance (SBA) | Routine delivery- Monitor labour with partograph, Use measures of infection prevention during delivery, measure blood pressure, controlled cord traction, oxytocin within 1 minute of delivery, uterine massage, place baby on mother’s abdomen after delivery etc.  Emergency obstetric care- Parenteral antibiotic, parenteral oxytosin, parenteral anticonvulsant, manual removal of placenta, instrumental delivery etc.  Emergency newborn care- Injectable antibiotics for newborn sepsis, newborn resuscitation with bag and mask, skin-to-skin or Kangaroo Mother Care for low birth weight etc.  Non-medical quality- Woman can choose to have delivery companion, patient toilet exists, toilet has water for hand washing, toilet is clean, toilet has soap for hand washing. |
| Lozano et al., 2006 [[6](#_ENREF_6)] | Process & outcome | ANC | Received blood test and had BP measured |
|  |  | SBA | Birth took place in hospital |
|  |  | Services delivered to premature babies | Difference in mortality rate in premature babies compared with max and min risk-adjusted mortality |
|  |  | ARI | Treatment from a health worker |
| Okawa S. et al., 2019 [[7](#_ENREF_7)] | Process | ANC | Tetanus toxoid two doses injected, blood pressure measured, deworming, vitamin b1 tablets given, iron folate tablets prescribed, HIV tested, syphilis tested, urine protein checked, tuberculosis screening done, body weight measured, urine sugar checked, emotional status, haemoglobin, domestic violence checked |
|  |  | Peripartum care | Newborn body dried, disposable delivery kit used, delivered on a clean floor/bed, first bathing of newborn after 6 hours, birth weight measured, breastfeeding initiated <30 min, skin-to-skin contact |
|  |  | Postnatal care | Maternal- family planning counselling, blood pressure measured, temperature measured, anaemia checked, iron folate prescribed, Vitamin B1 tablets, Vitamin A tablets, breast and nipple checked, vaginal healing checked, uterus checked, lochia checked, emotional status checked.  Neonatal- BCG immunisation given, Hepatitis B immunisation, temperature measured, physical examination, breast feeding checked, |
| Yakob et al., 2019 [[8](#_ENREF_8)] | Process | Family planning | Discussed STI prevention and condom, partner status, asked STI symptoms, checked current breastfeeding, asked chronic illness, discussed HIV risks, assured confidentiality, used visual aids, asked desired timing of next chid, assed menstrual regularity, asked last deliver date, asked client age, asked reproductive intentions, Measured blood pressure etc. |
|  |  | ANC | Checked blood pressure, measured weight, checked fetal heartbeat, checked uterine height, checked pallor, asked LMP, done HIV test, done urine test, done anaemia test, counselled nutrition, asked danger signs, provided TT vaccination, HIV counselling, syphilis test, provided iron etc. |
| Wang et al., 2019 [[9](#_ENREF_9)] | Structure | Facility delivery | Domain A: Comprehensive emergency obstetric care  Parenteral administration of antibiotics, parenteral administration of uterotonic drugs/oxytocin, Parenteral administration of anticonvulsants, manual removal of placenta, assisted vaginal delivery, removal of retained products, caesarean section, blood transfusion  Domain B: Newborn signal functions and immediate care  Neonatal resuscitation, skin to skin, breastfeeding in 1st hour, drying and wrapping newborns  Domain C: General requirements  Improved water source, electricity, improved sanitation, 24/7 SBA, emergency transport  Domain D: Equipment  Sterilization equipment, delivery bed, examination light, delivery pack, Suction apparatus, manual vacuum extractor, partograph, gloves, newborn bag and mask, infant scale, blood pressure apparatus, disinfectant etc.  Domain E: Medicines and commodities  Hydrocortisone, injectable antibiotic, injectable uterotonic, skin disinfectant, magnesium sulphate, chlorhexidine for cord cleaning , antibiotic eye ointment, IV solution with infusion set  Domain F: Guidelines, staff training and supervision  Integrated Management of Pregnancy and Childbirth guidelines, EmOC Guidelines, guidelines for management of preterm labor,Training in neonatal resuscitation, training in early and exclusive breastfeeding, training in newborn infection management, training in cord care, training in CEmOC , supervision etc. |
| Leslie HH, et al., 2017 [[10](#_ENREF_10)] | Process | ANC | History taking- Last menstrual period to calculate gestational age, prior pregnancy experience, danger signs in current pregnancy, previous complications on record  Routine Examination – Provider assessed fundal height, weight, edema, vaginal exam, fetal heart rate, BP, ultrasound  Screening- Provider screened HIV, anaemia, syphilis test, blood group test, urine test  Preventive measures -Prescribed or gave iron or folic acid or both, provider prescribed or gave tetanus toxoid injection, prescribed or gave intermittent preventive treatment in pregnancy.  Education – Provider counselled on: Nutrition Sleeping under an insecticide-treated net, delivery planning: preparation (money, transport) and location emergency planning: supplies for home delivery Breastfeeding, post-partum and PNC, Pregnancy spacing  Record keeping- Provider completed ANC card |
|  |  | Family planning | Reproductive history- Age, living children, last delivery date, pregnancy complications, last menstrual period, desire for child / more children, desired timing for birth of next child, breastfeeding, menses  Health history/exam- Blood pressure, weight, smoking, STI symptoms, chronic illness, pelvic exam  Counselling on methods - Any counselling on method |
|  |  | Sick child care | History taking- Inability to drink anything, normal feeding pattern, sick feeding pattern, cough or difficult breathing, diarrhoea and blood in stool (dysentery), fever, vomiting , convulsions , maternal HIV status, ear problems  Routine examination – Weight, plotted weight on chart, temperature, pallor, edema of feet, mouth  Drug administration and immunization- Checked immunization card or immunized, Vitamin A dosage, deworming medication  Client Education and Counselling - Explained how to administer prescribed medication, directions for feeding , described danger signs requiring return to facility , scheduled/discussed return visit, gave diagnosis |
| Venkateswaran et al., 2019 [[11](#_ENREF_11)] | Process | ANC | Screening for hypertension, SFH measurement, screening for anaemia, antenatal ultrasound, screening for gestational diabetes mellitus, screening for asymptomatic bacteriuria, screening for Rh-type, screening for tetanus immunization status |
| Murphy et al., 2018 [[12](#_ENREF_12)] | Process & structure | Inpatient neonatal care | (I) Documentation of newborn characteristics- Age, sex, mode of delivery, Apgar score etc.  (II) Documentation of signs and symptoms- Temperature, bulging fontanelle, can suck or breastfed etc.  (III) Evidence of monitoring – weight, vital signs etc.  (IV) Correct antibiotic dose  (V) Correct oxygen treatment and (VI) Correct fluids and feeds prescribed  Structural domains:  (i) Infrastructure (three items), (ii) laboratory services (10 items), (iii) hygiene equipment (14 items), (iv) safe delivery equipment and drugs for mothers (37 items), (v) resuscitation equipment for newborns on the delivery ward (20 items), (vi) essential equipment in the newborn unit (NBU) (18 items), (vii) intravenous fluids and feeds in the NBU (eight items) and (viii) NBU drugs (17 items) |
| Marchant et al., 2015 [[13](#_ENREF_13)] | Process | ANC | Measured weight, measured height, measured blood pressure, urine test, HIV test, counselling on breast feeding, danger signs and birth preparedness. |
|  |  | SBA | Received active management of third stage of labour components |
|  |  | Postpartum check | Checked breasts, checked bleeding, counselling on danger signs, family planning and nutrition |
|  |  | Postnatal check | Checked weight, checked cord, examined danger signs, caregiver counselling on thermal care, counselling on breastfeeding |
| Koulidiati J-l, et al. , 2018 [[14](#_ENREF_14)] | Process & structure | Care seeking for childhood illness | Provider asks- danger signs, fever, cough, ear problems  Provider checks- child weight, temperature, anaemia, vaccination status  Provider knowledge on appropriate first-line management processes of (1) severe dehydration in a 2-year-old (five process indicators), (2) breathing difficulties in a 1-year-old (three process indicators), and (3) lethargy in a newborn (three process indicators) assessed by the three vignettes.  Availability of electricity, water, sanitation, transport, and waiting room |
| Hodgins et al., 2014 [[15](#_ENREF_15)] | Process | ANC | Blood pressure measurement, tetanus toxoid, ANC at < 4 months of gestation, urine testing, counselling on danger signs, HIV testing, iron supplement etc. |
| Joseph et al., 2020 [[16](#_ENREF_16)] | Process | Nutrition interventions | Provision of iron-folic acid (IFA) supplements and counselling on their side effects, counselling on appropriate nutrition and diets during pregnancy, and counselling and support for early and exclusive breastfeeding  Direct observation of breastfeeding initiation within 1 hour of delivery, the newborn being placed skin-to skin if breathing, and keeping the mother and newborn in the same room. |
| Willey et al., 2018 [[17](#_ENREF_17)] | Structure | SBA | Infrastructure- electricity, water  Infection prevention- disinfectants, glove, sterilizer  Monitoring labour- BP cuff, timer, stethoscope etc.  Essential drugs- Parenteral antibiotics, anticonvulsants etc.  Neonatal resuscitation- bag and mask  Clean cord care- sterile cord cutter, cord tie |
| Carter et al., 2018 [[18](#_ENREF_18)] | Structure | Care seeking for childhood illness | Diagnostics- Malaria Diagnostic (RDTs or microscopy), malnutrition diagnostic (MUAC or scale + height board + growth chart), ARI diagnostic (stethoscope or respiratory timer), General microscopy  Basic medicines- ORS, zinc, Artemisinin combination therapy (ACT), oral antibiotic  Severe/complicated illness medicines- Intravenous fluids, injectable quinine or artesunate, injectable antibiotics  Human Resources- Training, guidelines, supervision  Available services- Diagnosis and treat malaria, diarrhoea, ARI, malnutrition, facilitated referral capacity  Knowledge- Average performance on case scenarios |
| Munos et al., 2018 [[19](#_ENREF_19)] | Process & structure | ANC, delivery car, care-seeking for chid illness, postnatal care | Identified items of structural quality in the domains of service availability; availability of drugs, diagnostics, and commodities; and training, supervision, and availability of guidelines, |
| Martínez S. et al., 2011 [[20](#_ENREF_20)] |  | Breastfeeding | The presence of diarrheal disease or ARI |
| Leslie et al., 2019 [[21](#_ENREF_21)] | Outcome | ANC | Proportion of births at full term |
|  |  | Delivery care | Proportion of deliveries without complications or death. |
|  |  | Newborn care | Proportion of live births reaching 28 days without death due to respiratory infection, nosocomial infection or sepsis. |
|  |  | Diarrhoea management | Visits to IMSS family medicine clinics that did not result in hospitalization due to diarrhoea for children under age 5. |
| Engle-Stone et al., 2015 [[22](#_ENREF_22)] | Biomarkers | Nutrition | Vitamin A intake |
| Kyei et al., 2012 [[23](#_ENREF_23)] | Process | ANC | Weight measurement, height measurement, blood pressure measurement, urine sample taken for analysis, blood sample taken for analysis, offered voluntary counselling and testing, iron supplementation provided, antimalarial drug provided, birth preparedness plan discussed, treatment provided for intestinal parasites and tetanus toxoid vaccination. |
| Travassos et al., 2016 [[24](#_ENREF_24)] | Biomarkers | Immunization | Serum IgG antibodies to vaccine antigens |
| Colson et al., 2015 [[25](#_ENREF_25)] | Biomarkers | Immunization | Dried blood sample assay for measles-specific IgG |
| Gutiérrez, 2013 [[26](#_ENREF_26)] | Process | ARI | Treatment from a health worker |
|  |  | Delivery | Birth took place in hospital |
|  |  | Prenatal care | Received blood test and has blood pressure measured |
| Idzerda et al., 2011 [[27](#_ENREF_27)] | Process | ARI | The proportion of children with an ARI that received the correct treatment for this condition. |

ANC= Antenatal care, ARI= Acute respiratory infection,DPT= Diphtheria, pertussis, tetanus, ITN= Insecticide treated net, NBU= New born unit, ORS= Oral rehydration solutions, ORT= Oral rehydration therapy, SBA= Skilled birth attendance, STI= Sexually transmitted infection

**References**

1. Hategeka C, Arsenault C, Kruk ME: **Temporal trends in coverage, quality and equity of maternal and child health services in Rwanda, 2000–2015**. *BMJ global health* 2020, **5**(11):e002768.

2. Nguhiu PK, Barasa EW, Chuma J: **Determining the effective coverage of maternal and child health services in Kenya, using demographic and health survey data sets: tracking progress towards universal health coverage**. *Tropical Medicine & International Health* 2017, **22**(4):442-453.

3. Nguyen PH, Khương LQ, Pramanik P, Billah SM, Menon P, Piwoz E, Leslie HH: **Effective coverage of nutrition interventions across the continuum of care in Bangladesh: insights from nationwide cross-sectional household and health facility surveys**. *BMJ open* 2021, **11**(1):e040109.

4. Larson E, Vail D, Mbaruku GM, Mbatia R, Kruk ME: **Beyond utilization: measuring effective coverage of obstetric care along the quality cascade**. *International Journal for Quality in Health Care* 2017, **29**(1):104-110.

5. Nesbitt RC, Lohela TJ, Manu A, Vesel L, Okyere E, Edmond K, Owusu-Agyei S, Kirkwood BR, Gabrysch S: **Quality along the continuum: a health facility assessment of intrapartum and postnatal care in Ghana**. *PloS one* 2013, **8**(11):e81089.

6. Lozano R, Soliz P, Gakidou E, Abbott-Klafter J, Feehan DM, Vidal C, Ortiz JP, Murray CJ: **Benchmarking of performance of Mexican states with effective coverage**. *The Lancet* 2006, **368**(9548):1729-1741.

7. Okawa S, Win HH, Leslie HH, Nanishi K, Shibanuma A, Aye PP, Jimba M: **Quality gap in maternal and newborn healthcare: a cross-sectional study in Myanmar**. *BMJ global health* 2019, **4**(2):e001078.

8. Yakob B, Gage A, Nigatu TG, Hurlburt S, Hagos S, Dinsa G, Bowser D, Berman P, Kruk ME, Tekle E: **Low effective coverage of family planning and antenatal care services in Ethiopia**. *International Journal for Quality in Health Care* 2019, **31**(10):725-732.

9. Wang W, Mallick L, Allen C, Pullum T: **Effective coverage of facility delivery in Bangladesh, Haiti, Malawi, Nepal, Senegal, and Tanzania**. *PloS one* 2019, **14**(6):e0217853.

10. Leslie HH, Ndiaye Y, Kruk ME: **Effective coverage of primary care services in eight high-mortality countries**. *BMJ Global Health* 2017, **2**(3).

11. Venkateswaran M, Bogale B, Abu Khader K, Awwad T, Friberg IK, Ghanem B, Hijaz T, Mørkrid K, Frøen JF: **Effective coverage of essential antenatal care interventions: a cross-sectional study of public primary healthcare clinics in the West Bank**. *PloS one* 2019, **14**(2):e0212635.

12. Murphy GA, Gathara D, Mwachiro J, Abuya N, Aluvaala J, English M: **Effective coverage of essential inpatient care for small and sick newborns in a high mortality urban setting: a cross-sectional study in Nairobi City County, Kenya**. *BMC medicine* 2018, **16**(1):1-11.

13. Marchant T, Tilley-Gyado RD, Tessema T, Singh K, Gautham M, Umar N, Berhanu D, Cousens S, Schellenberg JRA: **Adding content to contacts: measurement of high quality contacts for maternal and newborn health in Ethiopia, north east Nigeria, and Uttar Pradesh, India**. *PloS one* 2015, **10**(5):e0126840.

14. Koulidiati J-L, Nesbitt RC, Ouedraogo N, Hien H, Robyn PJ, Compaoré P, Souares A, Brenner S: **Measuring effective coverage of curative child health services in rural Burkina Faso: a cross-sectional study**. *BMJ open* 2018, **8**(5):e020423.

15. Hodgins S, D'Agostino A: **The quality–coverage gap in antenatal care: toward better measurement of effective coverage**. *Global Health: Science and Practice* 2014, **2**(2):173-181.

16. Joseph NT, Piwoz E, Lee D, Malata A, Leslie HH, Group CCTW: **Examining coverage, content, and impact of maternal nutrition interventions: the case for quality-adjusted coverage measurement**. *Journal of global health* 2020, **10**(1).

17. Willey B, Waiswa P, Kajjo D, Munos M, Akuze J, Allen E, Marchant T: **Linking data sources for measurement of effective coverage in maternal and newborn health: what do we learn from individual-vs ecological-linking methods?** *Journal of global health* 2018, **8**(1).

18. Carter ED, Ndhlovu M, Eisele TP, Nkhama E, Katz J, Munos M: **Evaluation of methods for linking household and health care provider data to estimate effective coverage of management of child illness: results of a pilot study in Southern Province, Zambia**. *Journal of global health* 2018, **8**(1).

19. Munos MK, Maiga A, Do M, Sika GL, Carter ED, Mosso R, Dosso A, Leyton A, Khan SM: **Linking household survey and health facility data for effective coverage measures: a comparison of ecological and individual linking methods using the Multiple Indicator Cluster Survey in Côte d’Ivoire**. *Journal of global health* 2018, **8**(2).

20. Martínez S, Carrasquilla G, Guerrero R, Gómez-Dantés H, Castro V, Arreola-Ornelas H, Bedregal P: **Effective coverage of health interventions in Latin America and the Caribbean: metrics for the assessment of health systems performance**. *Salud publica de Mexico* 2011, **53**:s78-84.

21. Leslie HH, Doubova SV, Pérez-Cuevas R: **Assessing health system performance: effective coverage at the Mexican Institute of social security**. *Health policy and planning* 2019, **34**(Supplement_2):ii67-ii76.

22. Engle-Stone R, Nankap M, Ndjebayi AO, Vosti SA, Brown KH: **Estimating the effective coverage of programs to control vitamin A deficiency and its consequences among women and young children in Cameroon**. *Food and nutrition bulletin* 2015, **36**(3_suppl):S149-S171.

23. Kyei NN, Chansa C, Gabrysch S: **Quality of antenatal care in Zambia: a national assessment**. *BMC pregnancy and childbirth* 2012, **12**(1):1-11.

24. Travassos MA, Beyene B, Adam Z, Campbell JD, Mulholland N, Diarra SS, Kassa T, Oot L, Sequeira J, Reymann M: **Immunization coverage surveys and linked biomarker serosurveys in three regions in Ethiopia**. *PLoS One* 2016, **11**(3):e0149970.

25. Colson KE, Zúñiga-Brenes P, Ríos-Zertuche D, Conde-Glez CJ, Gagnier MC, Palmisano E, Ranganathan D, Usmanova G, Salvatierra B, Nazar A: **Comparative estimates of crude and effective coverage of measles immunization in low-resource settings: findings from Salud Mesoamérica 2015**. *PloS one* 2015, **10**(7):e0130697.

26. Gutiérrez JP: **Gaps in effective coverage by socioeconomic status and poverty condition**. *Salud publica de Mexico* 2013, **55**:s106-s111.

27. Idzerda L, Adams O, Patrick J, Schrecker T, Tugwell P: **Access to primary healthcare services for the Roma population in Serbia: a secondary data analysis**. *BMC international health and human rights* 2011, **11**(1):1-14.
